# The Evolution of Lithography: From Resolution Scaling to Manufacturing Constraints

**DOI:** 10.3390/mi17020261

**Published:** 2026-02-18

**Authors:** Heejoon Chae, Hyunje Park, Dae Joon Kang

**Affiliations:** 1 Department of Physics, Sungkyunkwan University, 2066, Seobu-ro, Jangan-gu, Suwon-si 16419, Gyeonggi-do, Republic of Korea; 2 Department of Science Education, Jeonju University, 235, Cheonjam-ro, Wansan-gu, Jeonju-si 55069, Jeonbuk-do, Republic of Korea

**Keywords:** nanolithography, nanofabrication, nanopatterning, lithography evolution

## Abstract

Lithographic patterning continues to evolve under the dual pressure of ever-finer features and manufacturable, cost-effective integration. Beyond headline resolution, industrial adoption is increasingly determined by a small set of coupled metrics: throughput, overlay (registration), defectivity, and cost, as well as by how these trade-offs shift with materials, substrate form factors, and integration flows. Here, we review lithographic techniques across three eras: traditional methods (pre-1990s), non-conventional innovations (1990s), and contemporary advancements (post-2000s), with an explicit goal that goes beyond compilation. Specifically, we provide a decision framework for interpreting each method using the same manufacturing-relevant criteria. For each class of technique, we summarize the operating principle and representative process routes, then map the dominant bottlenecks to the metric that ultimately limits scale-up. This cross-cutting lens clarifies why many emerging methods are compelling at the physics level yet remain constrained at the system level, where process windows, in-line control, and compatibility with existing fabrication ecosystems govern viability. By connecting mechanism-level innovation to manufacturing-level constraints, this review offers practical guidance for researchers and engineers seeking to position nanolithography options for applications ranging from high-volume semiconductor production to agile prototyping and materials- or substrate-limited devices.

## 1. Introduction

As transistors continue to scale down to the nanoscale to improve computing performance and energy efficiency, advances in lithography have played a central role in enabling major breakthroughs in semiconductor manufacturing [1,2,3,4,5,6]. To meet increasing requirements for resolution, pattern placement accuracy, scalability, and manufacturability, a wide range of patterning approaches has been developed. Importantly, however, industrial adoption is rarely dictated by resolution alone. In practice, manufacturable patterning is governed by coupled metrics such as throughput, overlay (registration), defectivity, and cost, and by how these trade-offs evolve with materials, substrate form factors, and integration flows. In parallel, the ability to fabricate nanometric structures has accelerated progress in materials science and engineering. Accordingly, lithographic tools have become key enablers for the miniaturization and integration of electronic devices [6,7,8], healthcare equipment [9,10,11,12], biosensors [13,14,15], and metamaterials [16,17], underscoring the broad impact of nanofabrication. Against this backdrop, it is useful to view the evolution of lithography as a progression of manufacturing trade-offs, rather than as a simple sequence of inventions. With this lens, we organize lithographic techniques into three eras and track how the dominant scale-up bottlenecks have shifted over time.

### 1.1. Traditional Lithography (Pre-1990s)

Traditional lithography became the mainstream of semiconductor manufacturing from the 1960s to the 1990s and drove major progress in microelectronic device fabrication [18,19,20]. Photolithography (PL) has enabled high-throughput patterning with reliable overlay using mature tools and standardized process flows [21]. However, further scaling has been increasingly limited by resolution and pattern fidelity because diffraction limit and process-related constraints make it difficult to create smaller feature sizes [22]. As critical dimensions approached these optical limits, tightening process windows translated directly into higher defectivity risk and yield sensitivity, elevating the importance of process control and metrology in high-volume manufacturing (HVM) [23,24]. To push beyond the diffraction limit, electron beam lithography (EBL) and X-ray lithography (XRL) were developed to achieve higher-resolution patterning [25,26,27,28,29,30]. However, these techniques remained unsuitable for large-scale fabrication. EBL inherently suffers from low throughput due to the serial exposure process, while XRL requires complex source, optics, and mask infrastructure that increases the cost and makes fab integration difficult [11,31,32]. In general, traditional patterning methods not only require tedious and iterative steps but also represent the limitations in resolution, positional accuracy, scalability, and versatility, thereby resulting in low yield and high cost in the fabrication process [2,3,4,5,6,11,32].

### 1.2. Non-Conventional Lithography (1990s)

To address these challenges, non-conventional lithography expanded the toolkit for micro and nanoscale patterning by leveraging alternative physical and chemical mechanisms, with many approaches gaining strong momentum from the 1990s onward. Compared with traditional optical projection, these methods often trade tighter design control and registration for advantages in materials compatibility, replication-based throughput, or sub-optical feature formation.

Interference lithography (IL) enables parallel pattern formation over large areas using controlled optical interference, offering attractive throughput for periodic structures but limited pattern complexity and layout-level overlay flexibility [33,34]. Soft lithography (SL) provides low-cost, materials-compatible replication routes, favoring low-cost and gentle processing on flexible or bio-related substrates while facing challenges in high-precision overlay and defect control for dense, multilayer device integration [35]. Although nanoimprint lithography (NIL) easily achieves high-resolution patterning with an excellent throughput, its scalability highly relies on template defectivity, particle control, and repeatable overlay over large areas [36,37,38,39]. Nanosphere lithography (NSL) provides a simple and scalable route to periodic nanostructures through colloidal self-assembly, typically prioritizing low cost and large-area coverage over deterministic placement and design freedom [40,41]. Although block copolymer lithography (BCL) generates nanoscale patterns through a self-assembly process in a controllable manner, scaling up is strongly limited by defect suppression, long-range order, and reliable registration to device layouts [42,43,44]. Dip-pen nanolithography (DPN) uses an atomic force microscope tip to deposit molecular inks with high spatial precision, enabling customized patterns for specialized applications, but remains constrained by serial-writing throughput and tool complexity that raise cost for large-area manufacturing [45,46].

Collectively, these non-conventional approaches broaden the patterning design space, but their scale-up remains constrained by recurring integration challenges, including pattern complexity, defect control, overlay (registration), materials compatibility, and compatibility with established process flows. Consequently, their impact has been strongest in application-specific niches where these trade-offs are acceptable, rather than as universal solutions for high-volume manufacturing.

### 1.3. Contemporary Innovations (Post-2000s)

Since the 2000s, contemporary innovations have increasingly focused on pushing the limits of resolution, overlay (registration), and manufacturability through extensions of optical systems, near-field concepts, and digitally controlled or highly parallelized pattern generation [47,48,49,50,51]. In this era, the central question shifts from whether nanoscale features are achievable to whether they can be delivered with acceptable throughput, defectivity, and cost within existing integration flows.

Immersion lithography (IML) enhances optical resolution and process latitude by increasing the effective numerical aperture (NA) through a high-refractive-index liquid and remains attractive due to its favorable balance of throughput, overlay control, and ecosystem maturity, despite tightening process windows at advanced nodes [52,53]. Extreme ultraviolet lithography (EUVL) uses 13.5 nm radiation and reflective optics to enable advanced semiconductor patterning, and progress toward high NA extreme ultraviolet systems highlights the continued drive toward next-generation nodes [54,55,56,57]. Plasmonic lithography (PSL) exploits near-field enhancement associated with surface plasmons at metal–dielectric interfaces to achieve sub-diffraction patterning [58,59]. Digital maskless lithography (DML), frequently implemented with spatial light modulators such as digital micromirror devices, provides reconfigurable patterning without physical masks [60]. Multi-beam maskless lithography (MBML) aims to overcome the throughput bottleneck of single-beam direct writing through massively parallel electron-beam exposure, which allows practical direct-write routes for selected high-value applications [61,62,63]. Electrohydrodynamic lithography (EHL) creates microscale and nanoscale patterns through a high electric field to induce the film instability and alleviates material constraints in the patterning process [64].

Although these contemporary approaches have demonstrated strong potential, scalability, defectivity, cost efficiency, and integration remain critical challenges, and ongoing efforts continue to improve their performance and practical adoption. Viewed through manufacturing-relevant metrics, they illustrate a common theme: many routes can achieve impressive resolution, but only a subset can simultaneously satisfy throughput, overlay, defectivity, and cost targets required for broad industrial deployment.

### 1.4. Overview of the Review

**Figure 1:**
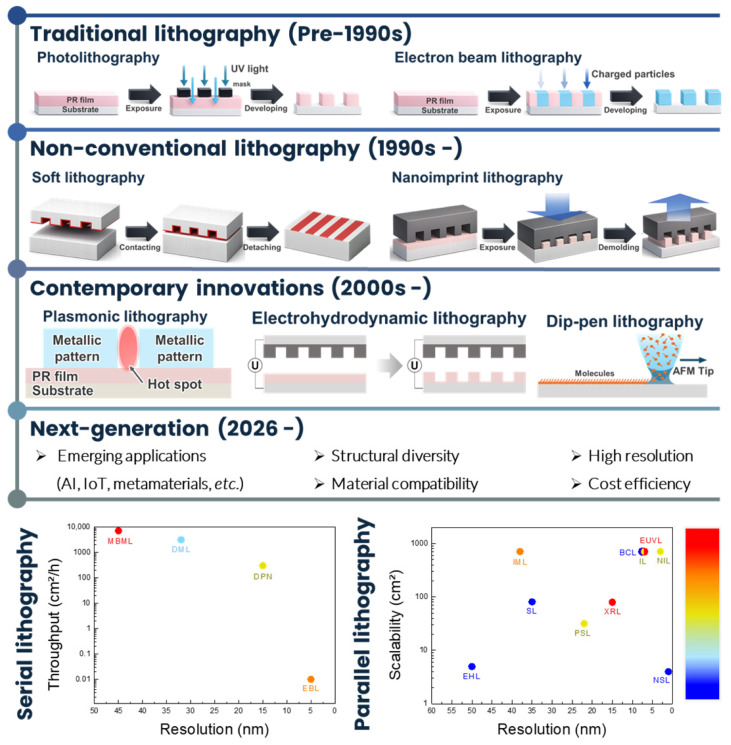
Chronological evolution of lithographic techniques. Patterning methods are classified into traditional, non-conventional, and contemporary innovations. Adapting recent trends for AI technology or IoT devices, next-generation lithography will be highly demanded. Inset plots summarize key trade-offs. The serial-lithography map (resolution–throughput–cost) compares serial techniques, with cost encoded by the color scale (low → high). The parallel-lithography map (resolution–scalability–cost) compares parallel techniques, again with cost represented by color [11,31,32,60,65,66,67,68,69,70,71,72].

This review provides a comprehensive overview of the rapidly evolving landscape of lithographic techniques. In contrast to prior reviews, we organize lithography into three eras, namely traditional lithography (pre-1990s), non-conventional lithography (1990s), and contemporary innovations (post-2000s). Figure 1 summarizes this chronological evolution and visually connects the three eras to emphasize how dominant bottlenecks and manufacturing trade-offs have shifted over time. By connecting these three groups, this review presents a holistic and forward-looking perspective on the continuous evolution of nanoscale patterning. To clarify practical applicability, the working principles of each approach are discussed, and key advantages and limitations are summarized with respect to manufacturing-relevant criteria, including throughput, overlay (registration), defectivity, and cost, as well as resolution, pattern fidelity, scalability, and process integration. Consistent with this decision framework, the inset plots in Figure 1 summarize the key trade-offs for serial and parallel patterning routes. Specifically, the serial-lithography map compares representative serial techniques in a resolution to throughput space with cost encoded by the color scale (low to high), whereas the parallel-lithography map compares parallel techniques in a resolution to scalability space, again with cost represented by color. In addition, we highlight major bottlenecks shared by many modern approaches, including defect control, cost efficiency, and manufacturability, and discuss the practical trade-offs required to balance scalability, cost effectiveness, and versatility.

Recent technological trends in artificial intelligence (AI), Internet of Things (IoT) devices, metamaterials, and wearable and bio-integrated systems continue to push requirements for next-generation lithography [73,74,75,76,77,78,79,80,81,82]. For example, the reliable fabrication of metamaterials and metasurfaces demands cost-effective patterning of intricate micro and nanoscale architectures, often with diverse inorganic materials and, in some cases, three-dimensional geometries [83,84]. These emerging requirements amplify the need for patterning strategies that can be compared on a common basis, because application viability is frequently determined by the coupled constraints of throughput, registration, defectivity, and total cost rather than by resolution alone. In this review, lithographic techniques are not compared solely by achievable resolution, but by whether they can simultaneously satisfy four manufacturing constraints: scalable throughput, reliable overlay and alignment, low defectivity within a practical process window, and cost predictability at scale. This perspective reframes nanolithography not as a resolution competition, but as a manufacturing discipline in which system-level integration ultimately determines viability. Unlike prior reviews that catalog lithographic techniques primarily by resolution or novelty, this work is explicitly structured to support decision-making under manufacturing constraints, addressing a gap between academic demonstrations and industrial adoption [11,31,32,60,65,66,67,68,69,70,71,72].

## 2. Traditional Lithography

Conventional lithographic methods have long underpinned semiconductor manufacturing and MEMS fabrication, enabling a broad range of micro and nanoscale devices. In the traditional era, photolithography established the high-volume benchmark by combining high throughput with reliable alignment and overlay control, yet continued scaling becomes increasingly constrained by diffraction-limited resolution and by the rising cost and process complexity associated with shorter exposure wavelengths, higher NA, and multipatterning. High-resolution alternatives such as electron beam lithography and X-ray lithography extend patterning capability beyond optical limits, but their industrial adoption is primarily restricted by manufacturing bottlenecks tied to throughput and cost, including serial write-time limits in EBL and the source, beamline, and mask infrastructure challenges in XRL. This section, therefore, introduces the basic principles, strengths, and limitations of PL, EBL, and XRL, and summarizes their dominant industrial bottlenecks and scalability-limiting parameters in Table 1. In Table 1, throughput is reported for serial methods, whereas scalability is reported for parallel methods.

### 2.1. Conventional Photolithography

**Figure 2:**
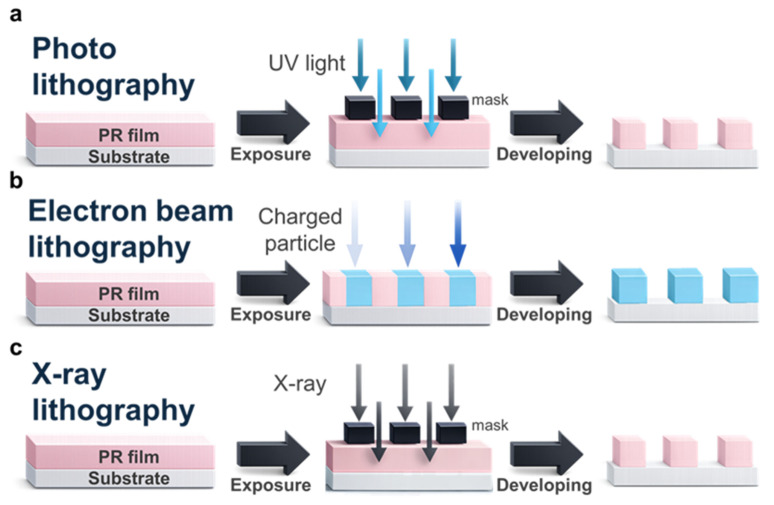
Schematic comparison of traditional lithographic processes. (**a**) PL uses a photomask and UV light to transfer the pattern onto a PR film on a substrate. (**b**) EBL uses a charged particle beam for direct writing on the PR surface without a mask. (**c**) XRL process utilizes an X-ray mask and X-ray exposure for patterning. These conventional methods require exposure and developing steps to generate patterned structures on the substrate.

Photolithography (PL) transfers a pattern from a photomask onto a photoresist (PR) film coated on a substrate through mask-based exposure and development steps (Figure 2a) [18,19,20]. First, a substrate, typically a flat, rigid wafer (e.g., silicon), is uniformly coated with a thin PR layer. The PR can be positive, where the exposed regions become soluble and are removed during development, or negative, where the unexposed regions are removed. The PR-coated substrate is then aligned to the photomask, and the pattern is transferred to the PR by UV-based optical exposure through projection optics. Historically, exposure sources have progressed from i-line UV (365 nm) to deep-UV systems such as KrF (248 nm) and ArF (193 nm) to support continued scaling, while further optical extensions such as immersion and EUV are discussed in later sections. Upon exposure, photochemical reactions modify PR solubility, and subsequent development selectively removes either the exposed or unexposed regions, yielding a patterned PR image. This resist pattern is then transferred to the underlying layers by etching or lift-off, completing the patterning sequence.

PL remains the dominant manufacturing platform because it offers high throughput and robust overlay control enabled by mature tooling and standardized process integration. However, its resolution and pattern fidelity are fundamentally constrained by diffraction and process-window limits, which become increasingly restrictive as critical dimensions shrink [18]. In practice, continued scaling often requires shorter wavelengths, higher NA optics, and more complex process flows, which in turn increase cost and tighten defectivity and yield sensitivity.

**Table 1:** Summary of traditional lithographic techniques.

**Technique**	**Strengths**	**Key** **Limitations**	**Industrial Bottleneck**	**Scalability-Limiting** **Parameter**	**Resolution** **(nm)**	**Throughput/** **Scalability**	**TRL**	**Ref**
Photo lithography (PL)	Mass production High throughput Alignment	Diffraction-limited resolution Multi-step flow	As finer patterns are required, the need for shorter exposure wavelengths, higher NA, and multipatterning drives a sharp rise in costs and process complexity.	Field size Stitching/overlay accumulation Focus/illumination uniformity	<1000	N/A- >700 cm^2^	8–9	[18,19,20]
Electron beam lithography (EBL)	Ultra-high resolution Maskless direct-write	Low throughput Proximity effect Beam instability	Although the resolution is excellent, the write speed (throughput) becomes a bottleneck, making it difficult to scale to large-area, high-volume manufacturing processes.	Dose per area Beam current Write time	<10	>0.01 cm^2^/h N/A	7–9	[27,85,86,87,88,89,90,91,92,93,94,95,96,97,98,99,100]
X-ray lithography (XRL)	Short wavelength Deep penetration	Source/beamline X-ray mask	It is difficult to supply high-performance X-ray sources and low-defect X-ray masks economically, which limits industrial adoption.	Source irradiance uniformity Mask defect density Mask flatness/thermal distortion	<20	N/A >50 cm^2^	3–6	[28,29,30,101,102,103,104,105,106,107,108,109,110]

Manufacturing bottleneck summary: PL is typically resolution and defectivity limited at advanced nodes, with cost escalating as process complexity and patterning requirements increase, while throughput and overlay remain relative strengths.

### 2.2. Electron Beam Lithography

Electron beam lithography (EBL) uses a focused charged-particle electron beam to directly write patterns on a PR film without a photomask (Figure 2b), enabling highly customized, high-resolution patterning [25,26,27]. In a typical process, a substrate is coated with an electron-sensitive resist such as polymethyl methacrylate (PMMA), with thickness chosen according to the targeted resolution and pattern-transfer requirements. A finely focused electron beam is then raster-scanned across the resist surface with an accelerating voltage typically in the range of 10 to 50 keV [111,112,113,114,115,116]. During exposure, the beam induces chemical changes in the resist, including chain scission in common positive resists (e.g., PMMA) and cross-linking in negative resists, thereby modulating solubility. Because the beam position, dose, and dwell time are digitally controlled from computer-aided design data, EBL provides maskless patterning with flexible layout updates compared with photolithography. After exposure, the resist is developed so that the exposed regions of a positive resist are removed, or the unexposed regions of a negative resist are removed, yielding a patterned resist image. The pattern is subsequently transferred to the underlying layers via etching, followed by resist removal to leave the final patterned structure [85,86,87,88].

EBL can achieve exceptional resolution, often below 10 nm, owing to the short de Broglie wavelength of electrons and the ability to focus the beam to a nanoscale spot [89,90,91,92,93,94,95]. However, its manufacturing scalability is fundamentally constrained by serial, point-by-point writing, which imposes a throughput ceiling and drives high cost per patterned area [96]. In addition, proximity effects from electron scattering, resist charging, thermal drift, and beam instability can degrade critical-dimension control and uniformity, increasing defectivity risk and complicating process control [32,87,88,97,98,99,100].

Manufacturing bottleneck summary: EBL is primarily throughput-limited by serial exposure and consequently cost-limited for large-area production, while overlay and defectivity are often constrained by proximity effects, tool stability, and resist charging during scale-up.

### 2.3. X-Ray Lithography

X-ray lithography (XRL) uses short-wavelength X-ray exposure in combination with an X-ray mask to transfer patterns onto an X-ray-sensitive resist (Figure 2c), offering a potential route to high-resolution patterning beyond conventional UV optics [28,29,30]. In a typical configuration, the mask comprises a thin X-ray-transparent membrane (e.g., silicon carbide or related materials) patterned with a high-contrast absorber that blocks incident X-rays to define the desired layout. The substrate is coated with an X-ray-sensitive resist, and X-rays transmitted through the mask selectively expose the resist, inducing chemical changes in the exposed regions. After exposure, the resist is developed to create a patterned resist image, which is then transferred to the underlying layers by etching, followed by resist stripping to reveal the final pattern.

The short wavelength and high penetration depth of X-rays enable reduced diffraction effects, supporting fine patterning and, in some implementations, thick, high-aspect-ratio structures [101,102,103,104,105,106]. However, despite its resolution potential, XRL has faced persistent manufacturing barriers that map directly onto cost, throughput, and integration complexity. High-performance X-ray sources and beamline infrastructure are difficult to supply and operate economically, and X-ray mask fabrication and handling introduce additional challenges, including mask defect density, mask flatness, and thermal distortion, all of which can degrade pattern fidelity and yield [107,108,109,110]. Source irradiance uniformity and the associated process window also become scalability-limiting factors as the patterning area increases. As a result, XRL has remained primarily confined to research and specialized niche applications, rather than being adopted as a mainstream high-volume manufacturing platform [30].

Manufacturing bottleneck summary: XRL is predominantly cost- and infrastructure-limited (sources, beamlines, and mask fabrication), with scalability additionally constrained by mask defectivity and stability and by source irradiance uniformity during large-area exposure.

## 3. Non-Conventional Lithography

Non-conventional lithography, introduced in the 1990s, encompasses diverse innovative techniques developed to overcome the practical constraints of traditional top-down lithography. In particular, to address two persistent issues, diffraction-limited resolution and escalating cost and process complexity, a broad set of alternative patterning routes was investigated during this period, and many continue to evolve today. By leveraging alternative physical, chemical, or mechanical mechanisms, non-conventional patterning methods expand the toolkit for micro and nanoscale fabrication and often enable materials-compatible and application-tailored patterning beyond conventional optical workflows. At the same time, their path to scale-up is typically determined by manufacturing-relevant bottlenecks, including throughput, overlay (registration), defectivity, and integration with established process flows. This section discusses six prominent non-conventional lithographic methods, IL, SL, NIL, BCL, NSL, and DPN, introducing their principles, strengths, and limitations, and summarizing their industrial bottlenecks, scalability-limiting parameters, and conceptual technology readiness levels in Table 2. In Table 2, throughput is reported for serial methods, whereas scalability is reported for parallel methods.

### 3.1. Interference Lithography

**Figure 3:**
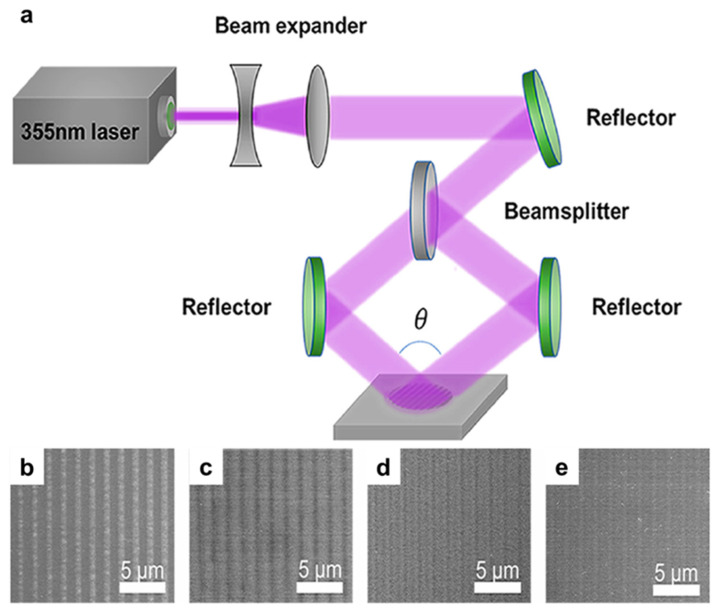
(**a**) The schematic image of the fabrication of periodic microstructures by laser interference. (**b**–**e**) SEM images of microstructures with laser fluences. Reprinted from Ref. [194], © 2020 MDPI (CC BY 4.0).

Interference lithography (IL), first explored for nanoscale patterning in the late 1990s, produces periodic structures in a photosensitive resist by exploiting the interference of two or more coherent laser beams [6,31,32]. As schematically shown in Figure 3a, an expanded 355 nm laser beam is split into two coherent beams and redirected by reflectors to intersect on the substrate at an angle *θ*, generating a spatially periodic intensity distribution [194]. The photoresist (or target surface) is selectively modified according to these interference fringes, enabling rapid formation of large-area periodic microstructures, with the pattern pitch primarily governed by the wavelength and intersection geometry. Representative SEM images (Figure 3b–e) illustrate how the resulting microstructure morphology and contrast vary with laser fluence, highlighting fluence as a practical knob for tuning feature definition and depth [194]. The achievable pitch and feature size are primarily dictated by the exposure wavelength and the interference geometry, particularly the angle between the interfering beams, and sub-50 nm periodic features can be obtained with appropriate optical configurations.

Because IL is a maskless, optical parallel process, it is well suited for large-area, high-throughput patterning of regular periodic structures at relatively low cost, making it attractive for diffraction gratings, photonic crystals, and other periodic nanostructures [117]. However, IL is intrinsically constrained in pattern versatility, because arbitrary and aperiodic layouts cannot be generated without additional optical complexity or hybrid process steps [125,126]. In addition, maintaining pattern fidelity over large areas requires precise alignment and stability of the optical setup, including beam coherence, vibration control, and dose uniformity, which can increase system complexity and cost [120,122,123,127,128,129,130].

Manufacturing bottleneck summary: IL is typically limited by overlay and pattern versatility for device-level integration, while throughput is a relative strength; defectivity and cost are governed by exposure stability and uniformity requirements during large-area processing. As a result, IL is best suited for large-area periodic structures such as gratings, photonic crystals, and metasurfaces, where layout complexity and multilayer overlay are not critical. Its adoption in device-level manufacturing that requires arbitrary pattern definition and precise layer-to-layer registration remains fundamentally limited.

### 3.2. Soft Lithography

**Figure 4:**
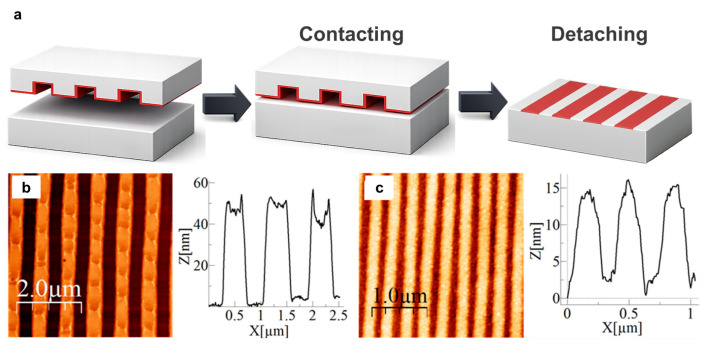
(**a**) Schematic representation of SL process in microcontact printing mode. (**b**,**c**) AFM images of the nanopattern fabricated by soft lithography. Reprinted from Ref. [195], © 2017 MDPI (CC BY 4.0).

Soft lithography (SL), developed in the early 1990s, uses flexible elastomeric stamps, such as polydimethylsiloxane (PDMS), to transfer patterns through conformal contact with a substrate [32]. Compared with rigid imprint-based processes, SL typically requires no high pressure or elevated temperature, making it a relatively low-energy and materials-compatible route for patterning. In practice, a soft stamp is used to replicate or print patterns via several modes, including microcontact printing [131,132,133], replica molding [134], and microtransfer molding [135]. In microcontact printing, an elastomeric stamp is first inked with molecular or nanoparticle “inks” and then brought into conformal contact with the substrate, transferring the ink only at the contacted regions (Figure 4a). Representative AFM images of the printed nanopatterns (Figure 4b,c) confirm faithful replication of periodic line features at the micro-/nanoscale, while the corresponding height profiles indicate well-defined topographic contrast after transfer [195]. Depending on stamp fidelity, ink chemistry, and process control, SL can achieve feature sizes on the order of tens to hundreds of nanometers, and it is technically compatible with curved or irregular surfaces. Because of this versatility, SL has been applied to flexible and unconventional substrates for applications including wearable electronics, microfluidics, and biomedical and bio-integrated systems [31,196,197].

Despite these advantages, SL faces intrinsic limitations in pattern fidelity and registration. Elastomeric stamps can deform during contact, leading to pattern distortion, linewidth variation, and reduced placement accuracy, particularly over large areas. Achieving finer features and better uniformity often requires high-quality master templates and tightly controlled stamp fabrication to minimize shrinkage, swelling, and mechanical creep. In addition, because SL relies on repeated contact between the stamp and substrate, practical manufacturing can be limited by contamination, ink transport nonuniformity, and stamp wear, all of which increase defectivity and degrade reproducibility over multiple cycles.

Manufacturing bottleneck summary: SL is most often limited by overlay and defectivity arising from stamp deformation and contact-induced variability, while cost and materials compatibility are relative strengths; throughput can be favorable for large-area replication when uniform contact and ink delivery are maintained.

### 3.3. Nanoimprint Lithography

**Figure 5:**
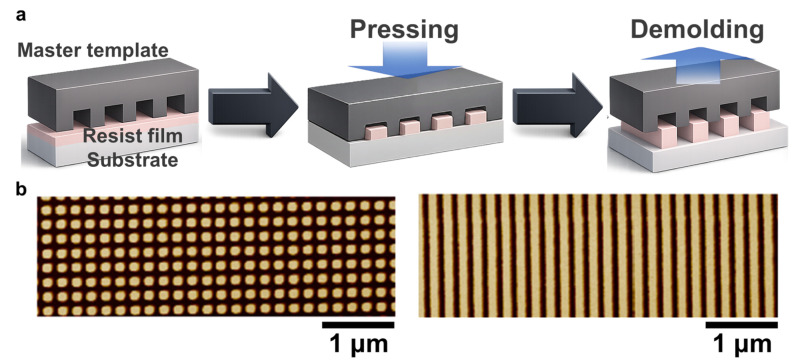
(**a**) Schematic representation of NIL process. (**b**) AFM data for the nanopatterns transferred by NIL process. Reprinted from Ref. [199], © 2024 MDPI (CC BY 4.0)

Nanoimprint lithography (NIL) transfers nanoscale features onto a resist-coated substrate by mechanically replicating a pre-patterned mold under applied pressure [37,38,39]. Compared with projection-based photolithography, NIL can be cost-effective because it avoids complex imaging optics, and it can achieve high resolution without electron-beam exposure during pattern transfer. The NIL process begins with the preparation of a mold, typically fabricated from rigid materials such as silicon or quartz, in which nanoscale features are defined using high-resolution patterning methods (often including EBL) and subsequent pattern-transfer steps. The target substrate is coated with a thin polymer resist layer, which can be either a thermoplastic polymer or a UV-curable formulation [38,136]. As schematically illustrated in Figure 5a, the mold is brought into conformal contact with the resist and pressed under controlled conditions. For thermoplastic resists, the system is heated above the glass transition temperature to soften the resist and allow mold features to be embossed. For UV-curable resists, UV exposure is applied while the mold is in contact to crosslink and solidify the resist. After solidification, the mold is released, yielding a negative replica of the mold topography in the resist.

NIL can achieve sub-10 nm resolution by relying on mechanical replication rather than optical imaging, thereby avoiding diffraction-limited constraints [138,139]. Because imprinting is inherently a parallel replication process, nanoimprint lithography (NIL) is attractive for large-area nanopatterning with high pattern fidelity [139,140,141,142,143]. As shown in the process schematic (Figure 5a), pattern transfer is achieved through conformal contact, pressing, and demolding, enabling rapid replication once a master mold is prepared. Representative AFM data (Figure 5b) highlight both the high-fidelity transfer and the practical sensitivity to defectivity, where particle contamination or trapped defects at the mold–resist interface can propagate into catastrophic pattern defects over large areas [198,199]. Repeated mold use can also lead to wear or damage that progressively degrades yield [37,136]. In addition, overlay (registration) across multiple layers can be challenging because mechanical contact, resist flow, and demolding may introduce distortion and nonuniform residual layers, particularly at high feature density. These issues can be further amplified on compliant or flexible substrates due to pressure-induced substrate deformation, necessitating specialized tooling and process optimization. Despite these challenges, NIL has been widely employed in nanophotonic devices [200,201], biomimetic studies [202,203,204], biosensors [205,206,207], and advanced optoelectronics, where controlled nanoscale sizes and shapes are often essential [137,199,208].

Manufacturing bottleneck summary: NIL is typically defectivity-limited by particle contamination and mold-related defects during contact replication, with overlay and distortion becoming critical for multilayer integration; cost can be favorable at scale, while throughput is a relative strength once defect and alignment control are established.

### 3.4. Block Copolymer Lithography

**Figure 6:**
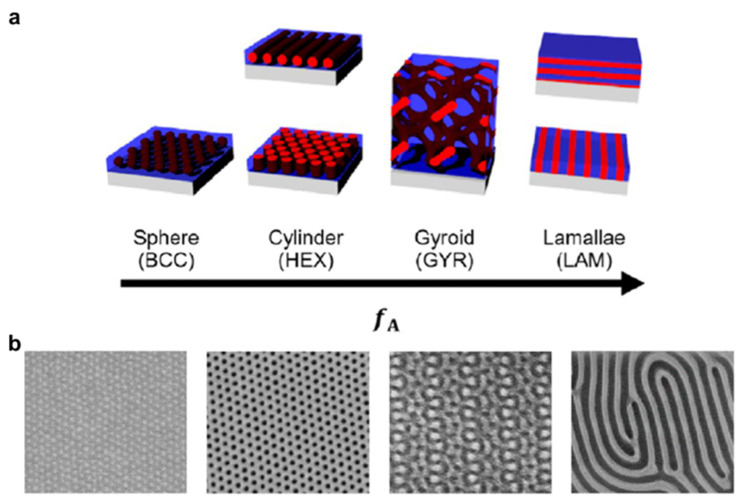
(**a**) Schematic illustration of block copolymer phase according to volume fraction ($f_{A}$). (**b**) SEM images show different phases of block copolymers. Reproduced with permission from Ref. [163]. © 2022 American Chemical Society.

Block copolymer lithography (BCL), introduced in the late 1990s, exploits the thermodynamic self-assembly of block copolymers to generate periodic nanoscale morphologies (Figure 6a) [43,144,145,146,147,148,149,150,151]. Block copolymers undergo microphase separation driven by segmental immiscibility and chain connectivity, producing ordered domains such as spheres and cylinders (Figure 6b) [152,153,154]. Thermal or solvent annealing promotes domain formation and ordering, and the resulting pitch and morphology are governed by molecular parameters including chain length, block volume fraction, and interaction strength [155,156,157,158,159,160]. After self-assembly, one block can be selectively removed or converted through etching, leaving a nanopatterned template that can be transferred to the underlying substrate.

By harnessing thermodynamically defined domain spacing, BCL can deliver feature sizes on the order of 10 nm, enabling applications in nanophotonics [152,153,161], advanced electronic pattern density extension concepts [162,163], and functional surfaces such as liquid-repellent re-entrant arrays [164] and antibacterial nanostructures [209]. This resolution advantage originates from microphase separation, where the characteristic pitch is set by molecular length scales rather than by optical imaging, making pattern formation intrinsically parallel and attractive for large-area nanopatterning.

At the same time, the same physics that enables parallel self-assembly also defines BCL’s practical scope. Self-assembly naturally generates periodic order, but it does not inherently provide deterministic, layout-defined placement of features across arbitrary device patterns. Consequently, BCL is best viewed not as a general replacement for layout-driven lithography, but as a density-multiplication or periodic-patterning strategy whose manufacturability is limited by registration and defect statistics at scale. Wafer-scale ordering is constrained by slow defect annihilation and kinetic trapping, which can leave dislocations, disclinations, orientation variability, and line-edge or line-width roughness that become yield-limiting as the patterned area increases. Directed self-assembly approaches using chemical or topographic guides can improve alignment and reduce defectivity, but they primarily shift the bottleneck to the quality and overlay of the guiding patterns and to narrow process windows, and they do not guarantee deterministic convergence to a uniformly low-defect state within manufacturing-relevant time scales [144,163]. In practice, this means that BCL is most compelling when the target pattern can tolerate periodicity and when the integration flow can supply high-quality guiding templates, rather than in applications that require arbitrary, placement-accurate layouts.

Manufacturing bottleneck summary: BCL is predominantly overlay- and defectivity-limited because deterministic placement and low-defect ordering are not intrinsic to self-assembly; throughput can be favorable due to parallel formation, but viable deployment depends on guide quality, process-window stability, and defect suppression at wafer scale. Therefore, BCL should be regarded as a density-multiplication or periodic-patterning strategy rather than a general replacement for layout-driven lithography. While sub-10 nm features are readily achievable, the lack of intrinsic deterministic placement and the difficulty of achieving wafer-scale, low-defect ordering with reliable registration fundamentally constrain its role in advanced semiconductor manufacturing.

### 3.5. Nanosphere Lithography

**Figure 7:**
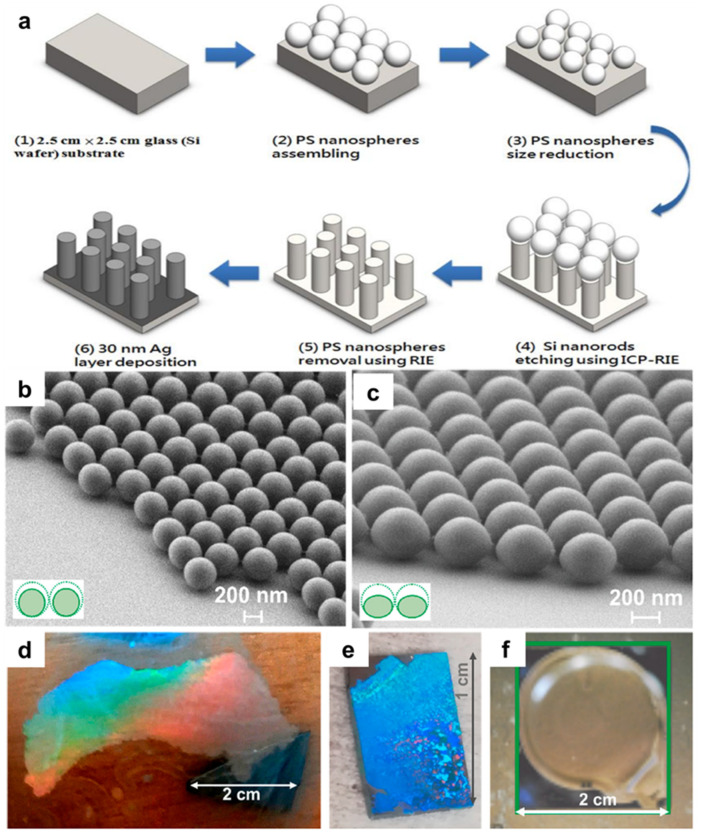
(**a**) Schematic diagram of the basic steps for creating ordered nanostructures through nanosphere mask. Reprinted from Ref. [210], © 2019 MDPI (CC BY 4.0) (**b**,**c**) Morphology of dispersed and etched nanospheres. The green inset schematics highlight the sphere shape change before and after etching. (**d**–**f**) Photographs of the PS-sphere monolayer on different surfaces. Reprinted from Ref. [211], © 2022 MDPI (CC BY 4.0).

Nanosphere lithography (NSL) is a bottom-up nanopatterning approach developed in the mid-to-late 1990s that uses the self-assembly of colloidal spheres to form an ordered mask for subsequent pattern transfer [40,41]. Nanospheres, commonly composed of silica, PMMA, or polystyrene (PS), can self-assemble into closely packed hexagonal monolayers on a substrate [41]. As schematically illustrated in Figure 7a, the assembled sphere array can be modified (e.g., by controlled sphere shrinking), followed by material deposition or etching, and subsequent sphere removal to yield periodically ordered nanostructures [210]. Figure 7b,c show representative morphologies of dispersed and etched nanospheres, highlighting how sphere packing and post-processing steps govern the effective mask geometry and, consequently, the transferred pattern. Photographs in Figure 7d–f further demonstrate large-area PS-sphere monolayers formed on different surfaces, underscoring the practical scalability of NSL beyond small coupons [211]. A key practical attraction of NSL is its simplicity and scalability, including demonstrations of large-area PS-sphere monolayers on the meter scale [41,124]. Because the sphere array functions as a reusable, parallel mask, NSL can generate a range of periodic nanostructures such as nanopillars, nanodots, and nanoholes with relatively low equipment complexity. Accordingly, NSL has been widely used in applications including surface-enhanced Raman scattering (SERS) [165,166], photonics [167,168], and plasmonics [169], where periodic nanostructures and large-area coverage can be more important than arbitrary layout definition.

Despite these strengths, NSL is best interpreted as a periodic, self-assembly defined patterning route rather than a general replacement for layout-driven lithography. Pattern quality is strongly dependent on the uniformity and long-range ordering of the nanosphere array, yet achieving reproducible monolayers requires tight control over sphere size distribution, solvent conditions, deposition parameters, and defect formation during assembly. In addition, pattern dimensions and placement are intrinsically coupled to sphere diameter and packing geometry, which limits deterministic control of feature arrangement and reduces design freedom for device-like layouts. These constraints motivate ongoing efforts to improve ordering control, defect suppression, and pattern tunability through modified assembly protocols, templated guidance, and hybrid integration schemes.

Manufacturing bottleneck summary: NSL is predominantly defectivity- and overlay-limited because long-range order and deterministic placement are not intrinsic to self-assembly; throughput and cost can be favorable due to parallel large-area masking, but scalability depends on reproducible monolayer formation and low defect density across the target area.

### 3.6. Dip-Pen Nanolithography

**Figure 8:**
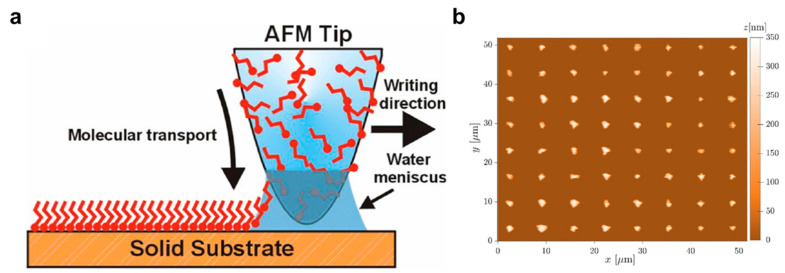
(**a**) Schematic representation of DPN process (**left**). Reproduced with permission from Ref. [192], © 2020 American Chemical Society. (**b**) AFM image of patterned surface (**right**). Reproduced with permission from Ref. [193]. © 2024 Wiley-VCH.

Dip-pen nanolithography (DPN) employs the tip of an atomic force microscope (AFM) as a nanoscale “pen” to directly deposit functional inks onto a substrate (Figure 8a) [46,172,173,174,175]. As schematically illustrated in Figure 8a, an inked AFM tip delivers material to the surface through a nanoscale meniscus mediated by surface tension and interfacial interactions, while the tip trajectory is controlled by a computer-guided positioning system [175,182]. The ink can consist of molecular precursors, nanoparticles, polymers, or biomolecules, enabling direct-write patterning with high compositional selectivity and placement control [177,178,179,180,181]. AFM imaging is frequently used to verify the written features and surface morphology (Figure 8b) [192,193]. DPN can achieve feature sizes on the order of tens of nanometers, and arrays of nanoscale dots can be printed with controlled spacing and size [176]. Because ink chemistry is highly tunable and substrates can be diverse, DPN is particularly useful for applications that require localized functionalization and customization, including biosensors [70,190] and selected quantum or nanoelectronic devices [191].

Nevertheless, DPN is fundamentally constrained by its serial writing nature, which imposes a severe throughput limitation and makes large-area manufacturing economically challenging. In addition, pattern reproducibility can be sensitive to environmental conditions such as humidity and temperature, because these factors affect ink transport, meniscus formation, and deposition kinetics, often necessitating controlled operating conditions [192,193].

Manufacturing bottleneck summary: DPN is primarily throughput-limited (serial direct writing) and therefore cost-limited for large-area patterning; defectivity and uniformity are strongly influenced by environmental sensitivity and ink transport stability, while overlay is generally strong for local placement within the tool’s positioning capability. Consequently, DPN is most appropriate for research, prototyping, and application-specific functionalization where pattern customization and material selectivity outweigh throughput considerations. Its extension to large-area or high-volume manufacturing remains economically impractical due to its intrinsically serial writing nature.

## 4. Contemporary Innovations

Since the early 2000s, lithographic methods have made great strides to effectively overcome the drawbacks of conventional and non-conventional nanopatterning techniques. In addition to extending established optical platforms, this period also introduced new patterning concepts that leverage near-field interactions, plasmonics, and field-driven film instabilities. By drawing on novel physical phenomena such as plasmonics, evanescent waves, and film instability, innovative nanopatterning approaches have been developed to create structurally diverse nanopatterns with low costs while improving the versatility and scalability. These contemporary innovations have broadened both device targets and integration contexts, enabling applications that span advanced semiconductor patterning, functional surfaces, and emerging form factors. However, ongoing studies continuously explore how to balance exceptional resolution with manufacturing-relevant requirements, including throughput, overlay (registration), defectivity, and cost, while maintaining compatibility with practical process integration. This section introduces several innovative patterning approaches with their basic mechanisms, benefits, and challenges. Table 3 provides a comparative overview of contemporary innovations in terms of strengths, key limitations, industrial bottlenecks, scalability-limiting parameters, and conceptual technology readiness levels (TRL).

### 4.1. Immersion Lithography

**Figure 9:**
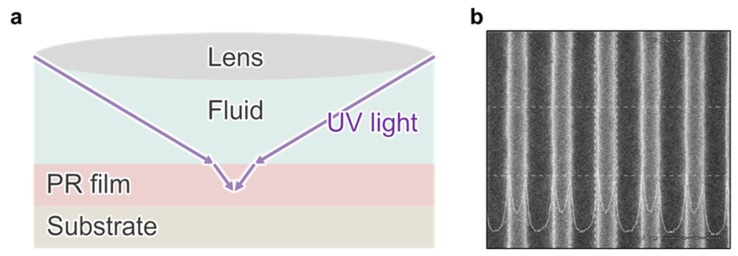
(**a**) Schematic representation of light traveling through lens, fluid, and PR film. (**b**) SEM image of 38 nm line array pattern transferred by IML process. Reprinted from Ref. [213], © 2022 MDPI (CC BY 4.0).

According to the definition of NA, NA = *n* sin (*θ*), increasing the refractive index (*n*) of the coupling medium enables a higher NA and thus improved optical resolution, motivating the development of immersion lithography (IML) in the 2000s. Utilizing this physical concept, IML improves resolution by inserting a high-refractive-index liquid (typically water in ArF immersion systems) between the wafer and the projection lens (Figure 9a) [52,53]. By raising the effective NA, immersion can enhance resolution while maintaining a usable depth of focus and process latitude, supporting continued device scaling. Leveraging the optical properties of the immersion fluid, IML has enabled the miniaturization of semiconductor devices, as exemplified by dense line array patterns transferred by the IML process (Figure 9b) [212,213]. Because it has enabled the fabrication of features smaller than 45 nm, IML has emerged as the industry standard for advanced semiconductor nodes. IML is highly effective in mass production because it builds on established photolithographic infrastructure and step-and-scan manufacturing flows [53]. However, this strategy faces practical limitations related to bubble and particle sensitivity, meniscus stability, and wafer-scale cleanliness, as well as the high cost of maintaining complex immersion systems, despite its widespread use [213,214,215]. Furthermore, as NA approaches its theoretical and materials-imposed limits, further scaling increasingly relies on system-level co-optimization, including tighter overlay control, defectivity suppression, and complex patterning strategies, rather than on NA increases alone [216,217].

Manufacturing bottleneck summary: IML remains strong in throughput and overlay, but defectivity and cost become increasingly limiting due to contamination and bubble sensitivity and the added complexity of immersion-specific hardware and process control.

### 4.2. Extreme Ultraviolet Lithography

**Figure 10:**
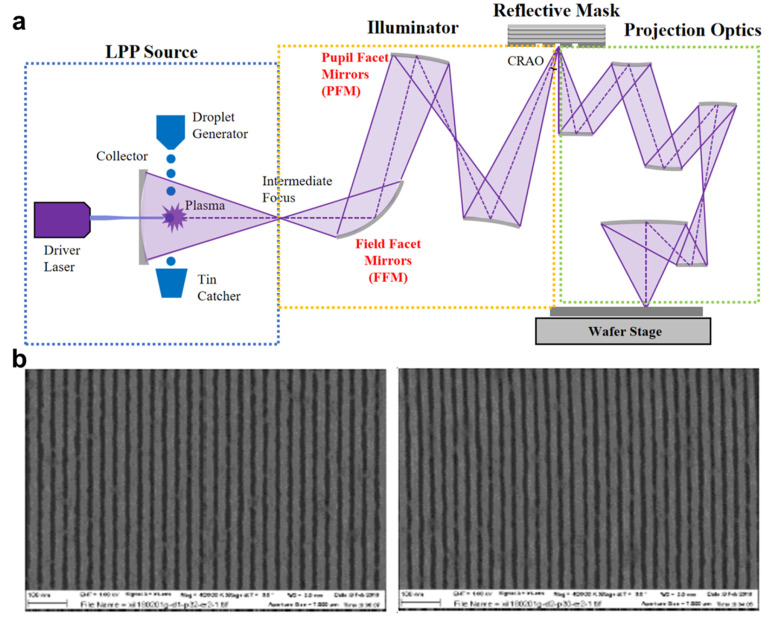
(**a**) Schematic image of the optical system for a EUVL process. Reprinted from Ref. [267], © 2025 MDPI (CC BY 4.0). (**b**) SEM images show the nanopatterns fabricated by EUVL (scale bar: 100 nm). Reproduced with permission from Ref. [268]. © 2018 American Chemical Society.

Progress in semiconductor manufacturing is intensively focused on reducing feature sizes to achieve faster and more advanced integrated circuits. This can be accomplished by lowering the exposure wavelength or increasing the NA of the projection lens in the system. Optical lithography has progressed through successive reductions in wavelength from 436 nm to 193 nm, followed by ArF immersion, to sustain continued node scaling [21,56]. Since the 2010s, extreme ultraviolet lithography (EUVL) using 13.5 nm wavelengths with the ultrahigh NAs has demonstrated the capabilities of reducing the resolution to below 10 nm, surpassing the capabilities of previously developed techniques. Instead of refractive lenses, reflected optical systems are utilized in the EUVL process due to the high EUV absorption of the equipment components (Figure 10a) [267]. The EUVL process requires a highly specialized EUV source, which is often a plasma created by powerful laser pulses that target tin droplets. A reflective mask coated with many thin layers of silicon and molybdenum is used to define the pattern, which is then projected onto a resist-coated substrate. Because of its ultrahigh resolution (Figure 10b), EUVL has played a vital role in developing semiconductor industries until now. Future integrated circuits and transistors benefit from its high precision and ability to design denser, smaller patterns [218,219,220,221,222].

However, industrial scalability is governed less by nominal resolution than by coupled constraints in throughput, defectivity, and cost. Source efficiency and delivered EUV power directly limit throughput, while complex reflective optics, vacuum infrastructure, and contamination control drive high capital and operating costs. In addition, mask and pellicle defects, along with stochastic resist effects, can translate into wafer-scale yield loss, making defectivity control a central challenge for manufacturing adoption [223]. Furthermore, EUVL still requires the development of alternative PR material capable of producing dense patterns at the sub-10 nm scale [220,222,224,225]. To resolve these challenges, many researchers have extensively investigated how to improve its processing efficiency [56,57,226,227,228,229,268,269,270,271].

Manufacturing bottleneck summary: EUVL is primarily constrained by cost and defectivity. Throughput is limited by available source power and system uptime, while yield is adversely affected by mask-related defects and stochastic resist phenomena at advanced nodes.

### 4.3. Digital Maskless Lithography

**Figure 11:**
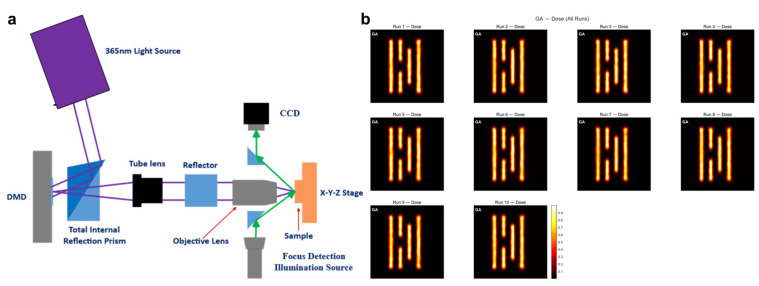
(**a**) Schematic representation of DML system. (**b**) Transferred line patterns. Reprinted from Ref. [272], © 2025 MDPI (CC BY 4.0).

In the traditional PL process, a physical photomask determines the patterning accuracy and quality. However, fabricating the photomask suitable for each patterning level is costly and technically challenging [230,231]. Motivated by this photomask issue, many efforts have been made to develop maskless lithography. Among these methods, maskless lithographic technique using digital light processing, known as digital maskless lithography (DML), has attracted great attention in the fields of industry and research. In the DML process, a pattern is initially prepared by design software and uploaded to the control system. The light for pattern transfer is followed by the trajectory of the uploaded pattern and accurately touches the patterning region. To achieve this, DML requires a digital micromirror device composed of many moving small mirrors, as illustrated in Figure 11a [231,272]. Depending on the tilted status and positions of mirrors, the incident light is properly guided to touch the targeted surface of the PR film [232,233]. Benefiting from this digital process, DML not only eliminates the need for expensive and time-consuming photomasks but also enables a customized patterning process and versatility. This mask-free, software-defined exposure is especially favored for application-driven manufacturing, where frequent design iterations and heterogeneous substrates can make mask-centric workflows inefficient. Especially, due to the recent advancements in AI technology, this digital patterning route will be continuously developing to further improve its lithographic capabilities.

Figure 11b shows representative transferred line patterns, demonstrating the feasibility of resist patterning through digitally controlled exposure [272]. Nevertheless, several drawbacks currently hinder broader adoption of DML. From a manufacturing perspective, DML is primarily constrained by throughput because exposure is typically performed over a limited field and must be stitched or scanned to cover large areas, which becomes considerably slower than high-volume mask-based projection. In addition, achievable resolution is strongly coupled to the DMD pixel size and projection optics, which can limit minimum feature size and pattern fidelity for advanced nodes [230]. When DML is applied to flexible or non-conventional substrates, the thermal budget of the resist and subsequent processing steps (e.g., baking/curing and post-exposure treatments) becomes an additional constraint that limits the usable process window.

Manufacturing bottleneck summary: DML is mainly throughput-limited for large-area production and can also be resolution-limited by pixel size; cost is favorable in low-to-medium volume use due to mask elimination, while overlay and defectivity increasingly depend on stitching accuracy and calibration as patterned area grows. Accordingly, DML is best positioned for rapid prototyping, low-to-medium volume production, and heterogeneous integration scenarios where design agility is critical. In these heterogeneous platforms, manufacturability is governed not only by exposure performance but also by low-damage, low-temperature process compatibility across the full patterning and transfer flow. In contrast, for cost-sensitive HVM, its scalability is constrained by throughput and stitching overhead as pattern complexity and area increase.

### 4.4. Multi-Beam Maskless Lithography

**Figure 12:**
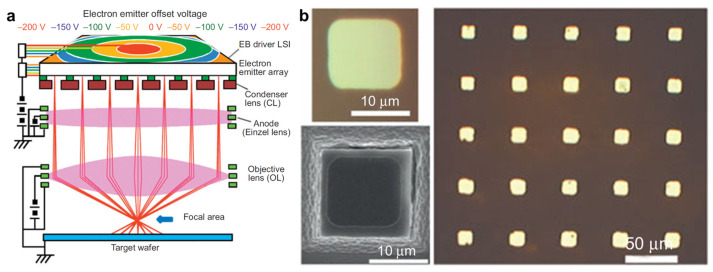
(**a**) Electron reduction optic system for MBML process. (**b**) Pattern image exposed by an arbitrarily selected 5 × 5 subset from the 200 × 200 electron emitter array. Reprinted from Ref. [273], © 2020 MDPI (CC BY 4.0).

Multi-beam maskless lithography (MBML) is an alternative patterning method using arrays of programmable electron beams or other focused energy sources, directly writing patterns onto a substrate without the need for conventional photomasks [61,62,63]. Unlike single-beam systems, this method achieves a very high resolution while greatly increasing throughput, making it ideal for fabricating intricate nanoscale structures [63,234,235,273]. A key enabler is the electron reduction optics that demagnify and project the emitter-array pattern onto the wafer through multi-element lens modules, allowing many beamlets to write in parallel within a defined focal field (Figure 12a) [273]. However, scaling to dense, stitched writing fields introduces stringent requirements on beam-to-beam uniformity, alignment, and aberration control; beamlet trajectories can exhibit distortion (e.g., field curvature and pincushion/barrel-type aberrations) without appropriate correction, directly impacting placement accuracy and pattern fidelity (Figure 12b) [273].

MBML is attractive for applications that benefit from maskless flexibility and high patterning precision, including rapid prototyping, specialized microelectronics, and mask fabrication, where design updates and short turnaround times are important [63,234,235]. At the same time, large-scale deployment is governed by system-level constraints that scale with parallelization. High system complexity and capital cost remain major barriers, and stable operation at high write rates requires uniform beam performance across the full array, long-duration calibration, and robust control of charging and thermal effects that can otherwise degrade pattern fidelity and defectivity. In practice, the scalability-limiting parameters often shift from beam physics to architecture-level factors, including the number of beams, per-beam write rate, data-path bandwidth, and long-term calibration stability.

Manufacturing bottleneck summary: MBML is primarily constrained by system complexity and cost, while throughput and defectivity at scale depend on uniform beam-to-beam performance, thermal and charging control, and calibration stability; overlay performance is increasingly limited by stage accuracy and multi-beam registration during high-area writing.

### 4.5. Plasmonic Lithography

**Figure 13:**
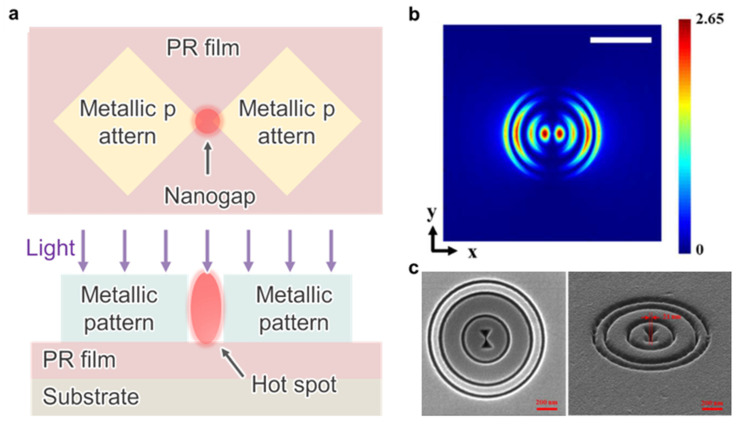
(**a**) Schematic design of PSL process setup. (**b**) Simulated results for locally enhanced electromagnetic field (scale bar: 500 nm). (**c**) SEM images of the nanosized structures obtained by PSL. Reprinted from Ref. [274] © 2023 MDPI (CC BY 4.0).

When an electromagnetic wave is incident on the metallic surface, collective electron oscillations are excited at the metal dielectric interface and generate a highly localized electromagnetic field, as depicted in Figure 13a,b [274]. Plasmonic lithography (PSL) employs this surface plasmon resonance to confine optical energy beyond the far-field diffraction limit during pattern transfer (Figure 13c) [58,59,274]. This physical origin of advantage enables pattern formation with resolutions down to approximately 10 nm and supports applications such as plasmonic sensors, photonic circuits, and advanced memory devices [236,237,238,239,240,241]. However, the physical origin of failure is inherent to the same plasmonic mechanism. Strong field confinement in common plasmonic metals is accompanied by substantial ohmic loss that converts optical energy into heat, which directly limits energy transmission efficiency and narrows the usable exposure window [242,243,244,245]. The resulting localized heating near plasmonic hotspots can distort pattern fidelity and degrade material stability in both the resist and the plasmonic structures, especially when repeated exposures or high-intensity operation are required [246]. This heat generation also makes PSL challenging to integrate with heterogeneous stacks and flexible/conformal substrates, where the thermal budget of non-conventional materials and interfaces is often limited. In addition, the near field decays rapidly with distance, so nanoscale variations in spacing, resist thickness, or surface planarity readily translate into critical dimension nonuniformity and reduced pattern integrity, which becomes increasingly problematic when scaling to large areas.

Engineering approaches, including hybrid patterning schemes, thermal management, and exploration of alternative low-loss plasmonic or polaritonic materials, can mitigate these effects. Nevertheless, as long as PSL relies on plasmonic confinement in practical materials, dissipation and hotspot-driven nonuniformity remain intrinsic constraints, and improvements often shift the dominant bottleneck to mask complexity, fabrication tolerance, and wafer-scale throughput. Therefore, expanding PSL beyond niche patterning scenarios would require exposure platforms and architectures that maintain nanoscale field control with uniformity while reducing dissipation-driven thermal loading in a manufacturable manner, for example, via genuinely low-loss material systems and scalable designs that avoid fragile, high-precision nanostructured masks [245,248,249,250]. In heterogeneous integration contexts, this requirement becomes even more stringent because thermal management needs to be achieved without exceeding the allowable thermal budget of temperature-sensitive layers.

Manufacturing bottleneck summary: PSL is primarily constrained by defectivity and scalability due to intrinsic, loss- and heat-driven limitations in process window and near-field uniformity. For temperature-sensitive materials, hotspot-induced heating can further tighten the process window and increase defectivity risk. Throughput and cost are further constrained by mask complexity and stringent fabrication tolerances required for large-area deployment.

### 4.6. Electrohydrodynamic Lithography

**Figure 14:**
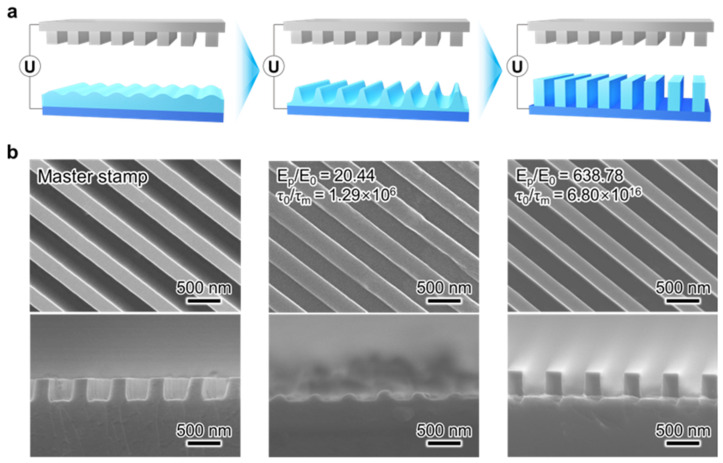
(**a**) Schematic representation of pattern transfer through EHL process, where *U* denotes the applied voltage and the blue and gray regions represent the polymer film and the master template, respectively. (**b**) SEM image showing the topology of the master pattern (**left**). Patterned features are controlled by electric field strength (**middle** and **right**). Reproduced with permission from Ref. [254], © 2023, American Chemical Society.

When a high electric field (>10^7^ V/m) is vertically imposed on the thin polymer film, electrostatic stresses destabilize the free surface and drive the formation of surface relief structures that reduce the electrostatic energy in a system [64,251,252,253]. This electrohydrodynamic instability provides a physical origin of advantage for pattern transfer because it can amplify nanoscale perturbations without relying on optical imaging and can therefore produce diverse micro and nanoscale topographies on a polymer surface, as represented in Figure 14a [254]. First, a spin-coated polymer layer is placed on a bottom electrode, and a master template as a top electrode is positioned above the film to create an air gap. When applying a voltage across two electrodes, the electrohydrodynamic instability is induced on the polymer surface and results in the pattern formation that reflects the field distribution defined by the template geometry (Figure 14b) [254]. Since its first report in the 2000s [53], EHL has been extensively studied owing to its rapid processing [254,255,256], the absence of diffraction-limited resolution constraints [65], structural diversity [257], and relatively simple equipment requirements [253,258,259,260,261]. Recently, several studies reported its extended applications, such as biomimetic structures [262] and biosensor devices [263], to further verify its versatility [258,260,264,265].

The practical positioning of EHL depends strongly on the target manufacturing regime. In practical terms, EHL is unlikely to serve as a manufacturing workhorse for advanced CMOS semiconductor integration, where deterministic overlay control, ultra-low defectivity, and wafer-scale uniformity within wide process windows are mandatory. This limitation arises not from insufficient resolution, but from the intrinsic sensitivity of instability-driven pattern formation to nanometric variations in gap spacing, electric field strength, and film thickness. At the same time, this does not preclude the use of electrohydrodynamic lithography in semiconductor-based devices more broadly defined, including oxide, organic, or flexible electronics, sensor arrays, and functional or energy-related device layers, where overlay tolerances are relaxed and materials compatibility, large-area coverage, and structural versatility become dominant requirements. For functional surfaces, bio-integrated devices, and flexible or materials-diverse platforms, EHL is especially attractive. It enables mask-defined topographic diversity, works well with soft materials, and supports rapid large-area patterning, which is useful when alignment does not have to be ultra-tight and heating must be kept minimal.

Manufacturing bottleneck summary: EHL is primarily constrained by defectivity and uniformity due to its intrinsic sensitivity to gap, field, and thickness variations, which limit its applicability to advanced CMOS manufacturing. However, for semiconductor-based functional devices and nontraditional substrates where deterministic overlay is not the dominant constraint, EHL remains a promising patterning platform owing to its materials compatibility, low-cost setup, and ability to generate structurally diverse micro- and nanoscale features within process flows that can be more compatible with low-thermal-budget materials [254].

## 5. Conclusions

Since the early 2000s, nanolithography has diversified rapidly, but the field’s central bottleneck has barely changed: industry does not reward resolution unless it arrives with throughput, overlay control, and predictable cost. The next decade will not be decided by who prints the smallest features in the lab, but by who can manufacture nanoscale patterns with repeatability, yield, and integration compatibility.

### 5.1. What Worked and What Did Not

In this review, we organized the development of nanolithography into three chronological tracks: conventional semiconductor lithography, non-conventional techniques that emerged in the 1990s, and contemporary innovations since the early 2000s.

Conventional platforms such as PL, EBL, and XRL matured together with the semiconductor industry since the 1980s. Their sustained industrial relevance stems from the fact that they can be engineered for large-scale process control, including alignment, overlay, and repeatable integration across multiple layers. However, continued scaling has been constrained by fundamental and practical barriers, including optical diffraction in PL, escalating tool complexity, and rapidly rising capital and operating costs.

Non-conventional approaches such as DPN, BCL, and NIL emerged as routes to bypass optical limits by exploiting new physical or chemical mechanisms. Despite decades of effort, many of these techniques have not translated broadly to high-volume manufacturing because they fail to satisfy at least one of the key manufacturing requirements. These requirements include high throughput, reliable overlay and alignment, low defectivity with high yield, and predictable cost at scale. In particular, methods that depend on delicate interfacial physics or multi-parameter self-organization often face narrow process windows, making wafer-scale uniformity and consistently low defect density difficult to guarantee.

Since the early 2000s, a new wave of innovations pursued resolution beyond the conventional optical roadmap. PSL and IML pushed optical patterning toward smaller features by taking advantage of near-field effects and higher refractive index media. In parallel, EHL demonstrated mask-defined pattern formation driven by electric field-induced instabilities. Maskless paradigms such as DML and MBML also expanded the design space by reducing dependence on photomasks. However, a recurring lesson from these developments is that improved patternability does not automatically translate into manufacturability. Many approaches remain limited by integration challenges such as overlay and multilayer registration, by throughput limitations inherent to serial or data-intensive writing, or by yield losses caused by defects and stochastic variation.

### 5.2. Where the Field Should Bet Next

**Figure 15:**
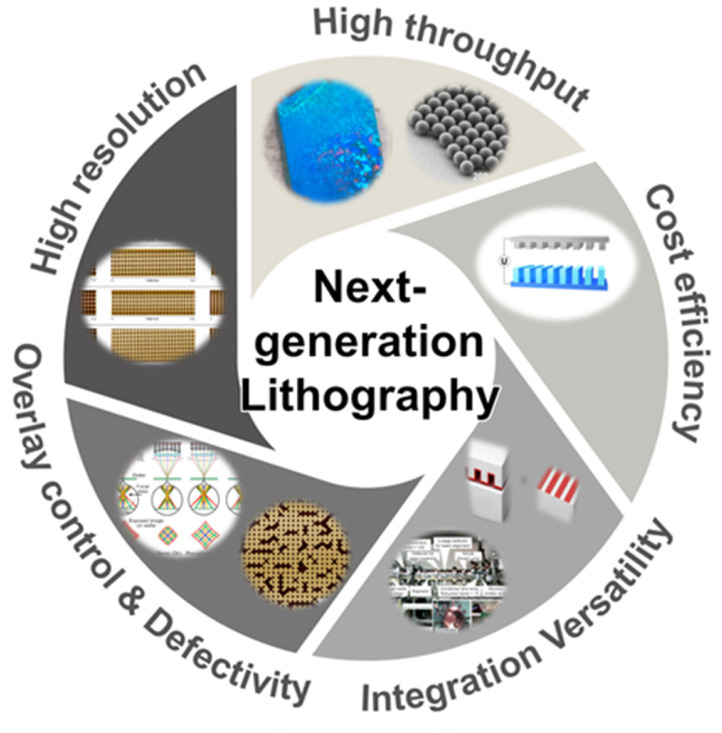
Conceptual summary of next-generation lithographic technologies evaluated by manufacturing-relevant criteria, including resolution, throughput, overlay control, defectivity, cost efficiency, and integration versatility. The figure emphasizes that only approaches satisfying all four manufacturing constraints simultaneously can serve as general-purpose platforms, while others are positioned as complementary or application-specific solutions. Importantly, placement along these axes reflects practical manufacturing outcomes reported across the literature, rather than idealized single-parameter demonstrations. Reprinted from Ref. [198], © 2021 MDPI (CC BY 4.0). Reprinted from Ref. [199], © 2024 MDPI (CC BY 4.0). Reprinted from Ref. [211], © 2022 MDPI (CC BY 4.0). Reproduced with permission from Ref. [254], © 2023, American Chemical Society. Reprinted from Ref. [273], © 2020 MDPI (CC BY 4.0).

The field should stop treating resolution as the primary metric and instead evaluate technologies by whether they satisfy four constraints simultaneously: (i) scalable throughput, (ii) overlay/alignment control, (iii) low defectivity with process windows, and (iv) cost predictability (Figure 15). Among the emerging techniques discussed in this review, only EUVL-class optical manufacturing currently satisfies all four constraints simultaneously for leading-edge semiconductor production, albeit at an extreme cost and system complexity. That uncomfortable fact explains why “cheaper high-resolution alternatives” routinely stall: they typically fail not at feature size, but at manufacturing integration. This does not mean EUVL is the final answer. It means that the next breakthroughs will not come from resolution alone, but from integration-first engineering.

At the same time, the dominant constraint set varies widely across product classes, and many applications are defined less by minimum feature size than by the ability to pattern reliably at the required manufacturing scale and cost. For leading-edge logic and memory, overlay, defectivity, and multi-layer process control remain decisive, favoring EUVL-class optical platforms. For dense periodic or templated structures, the practical question is whether ordering, defect suppression, and registration can be made deterministic enough to justify adoption, which naturally motivates self-assembly or imprint-based routes. For heterogeneous, large-area, or customization-driven manufacturing, throughput and design agility can outweigh ultimate resolution, creating space for maskless approaches and other non-conventional methods that trade peak performance for flexibility and lower mask dependence.

Concretely:

- BCL is a prime example of a technology that may be right scientifically yet still wrong industrially unless alignment becomes deterministic. Sub-10 nm features are not the barrier. The barrier is reliable long-range orientation, defect suppression, and registration to device layouts. If directed self-assembly cannot deliver a predictable overlay and low defectivity across large areas, it will remain best suited for templated or periodic patterns rather than serving as a general manufacturing platform.
- Maskless methods such as DML and MBML will win where design agility matters more than absolute throughput, including rapid prototyping, heterogeneous integration, and application-driven manufacturing. However, for cost-sensitive high-volume environments, serial writing will remain economically constrained unless parallelization and data path architectures advance faster than the growth of pattern complexity.
- EHL deserves separate emphasis because it offers a fundamentally different path to pattern formation. Rather than pushing optics to smaller wavelengths or relying on stochastic self-assembly, EHL converts an applied electric field into controllable surface instabilities that can amplify nanoscale perturbations and generate patterns that follow a template-defined field distribution. This mechanism makes EHL attractive for low-cost, large-area patterning and for creating structurally diverse topographies on polymer films. At the same time, EHL will not become a manufacturing workhorse unless it clears its own integration barriers, particularly uniformity across large areas, defect control, and repeatable process windows that remain stable against small variations in film thickness, air gap, and material rheology. If these sensitivities are engineered down through better process control and materials design, EHL could become one of the few routes that improve versatility without paying the full cost penalty of ultra-high-end optical systems. Importantly, the limitations of electrohydrodynamic lithography discussed here primarily apply to advanced CMOS logic and memory manufacturing. In application domains with relaxed overlay requirements and greater emphasis on materials diversity and form factor adaptability, EHL may offer a viable and complementary manufacturing route rather than a competing alternative to optical lithography.

Flexible and bio-integrated electronics represent a different constraint set in which the dominant requirement is not the minimum feature size, but materials compatibility, conformal contact, and pattern fidelity on curved or soft substrates. In addition, thermal budget constraints often become first-order considerations when non-conventional materials and hybrid stacks are involved. In these sectors, photomask-centric and rigid substrate workflows are often the wrong default. Accordingly, lithography paradigms may shift from maximizing resolution and overlay to enabling low-damage, low-temperature patterning with sufficient fidelity under curvature, roughness, and mechanical strain. Methods that tolerate curvature, roughness, and mechanical strain can dominate even at moderate resolution because they solve the system-level requirement. EHL is particularly relevant here because it operates naturally on thin polymer films and can generate patterns without direct mechanical contact, which may help preserve fragile or deformable substrates when the process is properly engineered.

Finally, machine learning and AI will matter most when used for manufacturing discipline rather than novelty. Their value is not in replacing physics-based understanding, but in tightening process windows into reproducible recipes by converting the most sensitive process degrees of freedom into measurable and controllable variables through accelerated defect inspection, parameter optimization, and tool drift compensation. In this review, the scalability-limiting parameters identified for each technique can be interpreted as high-sensitivity input features for data-driven models, including exposure dose and focus stability, overlay and stage drift, resist and film-thickness nonuniformity, gap and electric field sensitivity, and tool-to-tool variation. When coupled with in-line metrology and rapid defect inspection, such models can accelerate parameter optimization, enable early detection of excursions, and provide tool drift compensation to reduce defectivity and improve long-term process stability. Taken together, these tools reinforce the central premise of this review: practical success is determined less by peak resolution than by the ability to sustain stable, low-defect, and scalable manufacturing within realistic cost constraints.

In short, this manufacturing lens forces an uncomfortable truth. A lithography method can be scientifically elegant and still be industrially non-viable. The winners will be those who treat nanolithography not as a resolution contest, but as a manufacturing discipline in which cost, yield, overlay, and scalability determine what survives.

## Figures and Tables

**Table 2:** Summary of non-conventional lithographic techniques.

**Technique**	**Strengths**	**Key** **Limitations**	**Industrial Bottleneck**	**Scalability-Limiting** **Parameter**	**Resolution** **(nm)**	**Throughput/** **Scalability**	**TRL**	**Ref**
Interference lithography (IL)	Maskless Large-area periodic patterns	Periodic-only Limited arbitrary/complex patterns	Maintaining wafer-scale fringe stability (phase/coherence + vibration/thermal control) is the main hurdle for industrial use	Coherence/phase stability Vibration/thermal drift Fringe contrast uniformity	<10	N/A- >700 cm^2^	4–6	[33,34,117,118,119,120,121,122,123,124,125,126,127,128,129,130]
Soft lithography (SL)	Flexible/curved substrates Low cost	Stamp/mold deformation Master dependence	Large-area, high-volume replication is limited by stamp deformation and consistent pattern fidelity	Contact uniformity Swelling/solvent compatibility	<50	N/A >50 cm^2^	4–6	[31,35,131,132,133,134,135]
Nanoimprint lithography (NIL)	Sub-10 nm resolution Parallel replication	Particle/air trapping defects Template wear Release issues	Defect control (particles) and template lifetime/yield are the dominant barriers to HVM scaling	Pressure/gap uniformity Residual-layer uniformity	<10	N/A >700 cm^2^	6–8	[37,38,39,136,137,138,139,140,141,142,143]
Block copolymer lithography (BCL)	~10 nm natural pitch Large-area potential	Defect density Limited pattern diversity Long anneal	Achieving wafer-scale low-defect ordering with accurate alignment/registration is the key challenge	Ordering correlation length Guide-pattern uniformity	<10	N/A >700 cm^2^	4–6	[43,44,144,145,146,147,148,149,150,151,152,153,154,155,156,157,158,159,160,161,162,163,164]
Nanosphere lithography (NSL)	Low cost Simple process	Limited ordering control Low placement precision	Making defect-free, uniform monolayers over large areas with controlled ordering is the main bottleneck	Sphere monodispersity Monolayer uniformity	<10	N/A >4 cm^2^	4–6	[40,41,165,166,167,168,169]
Dip-pen nanolithography (DPN)	High precision Multi material Direct-write flexibility	Very low throughput Tip wear Ink diffusion/blur	Throughput is the fundamental limit for scaling to large-area manufacturing.	Write speed Tip-array parallelization	<20	300 cm^2^/h N/A	2–4	[46,70,170,171,172,173,174,175,176,177,178,179,180,181,182,183,184,185,186,187,188,189,190,191,192,193]

**Table 3:** Summary of contemporary innovations.

**Technique**	**Strengths**	**Key** **Limitations**	**Industrial Bottleneck**	**Scalability-Limiting** **Parameter**	**Resolution** **(nm)**	**Throughput/** **Scalability**	**TRL**	**Ref**
Immersion lithography (IML)	High resolution Infrastructure compatibility	Bubble/particle sensitivity Overlay complexity	At high NA, stable immersion meniscus control and wafer-scale cleanliness/overlay stability become increasingly difficult during fast scanning	Scan field Scan speed Meniscus stability Bubble/particle rate	<50	N/A >700 cm^2^	8–9	[52,53,212,213,214,215,216,217]
Extreme ultraviolet lithography (EUVL)	High resolution Leading-edge enablement	Tool cost Source power/uptime Resist sensitivity	Source power and uptime directly constrain dose-limited scan speed, while mask/pellicle defects and stochastic resist behavior impact wafer-scale yield	Source power Scan speed Defect yield Exposure time	<10	N/A >700 cm^2^	8–9	[218,219,220,221,222,223,224,225,226,227,228,229]
Digital maskless lithography (DML)	Mask-free Design flexibility	Limited resolution Low throughput	Resolution, projected field size, and throughput trade off against each other, and stitching overhead grows quickly for large-area patterns	Field size Stitching accuracy Step/scan speed	<50	>3000 cm^2^/h N/A	4–6	[230,231,232,233]
Multi-beam maskless lithography (MBML)	Massive parallelization Higher throughput	System complexity Beam uniformity	Uniform beam performance across the full array and stable long-duration calibration are required, while data streaming and charging/thermal control must remain stable at high write rates	Multi beams Per-beam write rate Long-term calibration	<50	>7000 cm^2^/h N/A	4–6	[61,62,63,234,235]
Plasmonic lithography (PSL)	Near-field super resolution	Plasmonic loss Hotspot heating	Large-area near-field enhancement is hard to keep uniform, and localized heating/loss can degrade materials and pattern fidelity	Near-field uniformity Gap/contact uniformity Thermal dissipation	<50	N/A >30 cm^2^	2–4	[58,59,66,236,237,238,239,240,241,242,243,244,245,246,247,248,249,250]
Electrohydrodynamic lithography (EHL)	Diffraction-free Simple setup Low cost Rapid patterning	Gap sensitivity Viscosity dependence Dielectric breakdown	Wafer-scale uniformity depends strongly on nm-level gap control and field/thickness uniformity, and breakdown/defect probability increases with patterned area	Gap/E-field/Film-thickness uniformity Breakdown probability	<50	N/A >5 cm^2^	3–5	[65,251,252,253,254,255,256,257,258,259,260,261,262,263,264,265,266]

## Data Availability

The raw data supporting the conclusions of this article will be made available by the authors on request.

## Abbreviations

The following abbreviations are used in this manuscript:

AFM	Atomic force microscope
AI	Artificial intelligence
BCL	Block copolymer lithography
DML	Digital maskless lithography
DPN	Dip-pen nanolithography
DUV	Deep ultraviolet
EBL	Electron beam lithography
EHL	Electrohydrodynamic lithography
EUV	Extreme ultraviolet
EUVL	Extreme ultraviolet lithography
HVM	High-volume manufacturing
IL	Interference lithography
IML	Immersion lithography
IoT	Internet of Things
MBML	Multi-beam maskless lithography
MEMS	Micro-electromechanical systems
NA	Numerical aperture
NIL	Nanoimprint lithography
NSL	Nanosphere lithography
PDMS	Polydimethylsiloxane
PL	Photolithography
PMGI	Polymethylglutarmide
PMMA	Poly(methyl methacrylate)
PR	Photoresist
PS	Polystyrene
PSL	Plasmonic lithography
SEM	Scanning electron microscope
SERS	Surface-enhanced Raman scattering
SL	Soft lithography
UV	Ultraviolet
XRL	X-ray lithography

## References

[1] Zhao H., Lee Y., Han M., Sharma B.K., Chen X., Ahn J.H., Rogers J.A. (2020). Nanofabrication Approaches for Functional Three-Dimensional Architectures. Nano Today.

[2] Luo S., Hoff B.H., Maier S.A., de Mello J.C. (2021). Scalable Fabrication of Metallic Nanogaps at the Sub-10 Nm Level. Adv. Sci..

[3] Chen Y., Shu Z., Zhang S., Zeng P., Liang H., Zheng M., Duan H. (2021). Sub-10 Nm Fabrication: Methods and Applications. Int. J. Extrem. Manuf..

[4] Oh D.K., Jeong H., Kim J., Kim Y., Kim I., Ok J.G., Rho J. (2021). Top-down Nanofabrication Approaches toward Single-Digit-Nanometer Scale Structures. J. Mech. Sci. Technol..

[5] Singh A., Shi A., Claridge S.A. (2022). Nanometer-Scale Patterning of Hard and Soft Interfaces: From Photolithography to Molecular-Scale Design. Chem. Commun..

[6] Sharma E., Rathi R., Misharwal J., Sinhmar B., Kumari S., Dalal J., Kumar A. (2022). Evolution in Lithography Techniques: Microlithography to Nanolithography. Nanomaterials.

[7] Bhagoria P., Sebastian E.M., Jain S.K., Purohit J., Purohit R. (2019). Nanolithography and Its Alternate Techniques. Mater. Today Proc..

[8] Barad H.N., Kwon H., Alarcón-Correa M., Fischer P. (2021). Large Area Patterning of Nanoparticles and Nanostructures: Current Status and Future Prospects. ACS Nano.

[9] Preetam S., Nahak B.K., Patra S., Toncu D.C., Park S., Syväjärvi M., Orive G., Tiwari A. (2022). Emergence of Microfluidics for next Generation Biomedical Devices. Biosens. Bioelectron. X.

[10] Greant C., Van Durme B., Van Hoorick J., Van Vlierberghe S. (2023). Multiphoton Lithography as a Promising Tool for Biomedical Applications. Adv. Funct. Mater..

[11] Stokes K., Clark K., Odetade D., Hardy M., Goldberg Oppenheimer P. (2023). Advances in Lithographic Techniques for Precision Nanostructure Fabrication in Biomedical Applications. Discov. Nano.

[12] Kammarchedu V., Asgharian H., Zhou K., Soltan Khamsi P., Ebrahimi A. (2024). Recent Advances in Graphene-Based Electroanalytical Devices for Healthcare Applications. Nanoscale.

[13] An T., Wen J., Dong Z., Zhang Y., Zhang J., Qin F., Wang Y., Zhao X. (2023). Plasmonic Biosensors with Nanostructure for Healthcare Monitoring and Diseases Diagnosis. Sensors.

[14] Fruncillo S., Su X., Liu H., Wong L.S. (2021). Lithographic Processes for the Scalable Fabrication of Micro- And Nanostructures for Biochips and Biosensors. ACS Sens..

[15] Dadras-Toussi O., Khorrami M., Louis Sam Titus A.S.C., Majd S., Mohan C., Abidian M.R. (2022). Multiphoton Lithography of Organic Semiconductor Devices for 3D Printing of Flexible Electronic Circuits, Biosensors, and Bioelectronics. Adv. Mater..

[16] Zheng H., Zhou Y., Ugwu C.F., Du A., Kravchenko I.I., Valentine J.G. (2021). Large-Scale Metasurfaces Based on Grayscale Nanosphere Lithography. ACS Photonics.

[17] Kagias M., Lee S., Friedman A.C., Zheng T., Veysset D., Faraon A., Greer J.R. (2023). Metasurface-Enabled Holographic Lithography for Impact-Absorbing Nanoarchitected Sheets. Adv. Mater..

[18] Levenson M.D., Viswanathan N.S., Simpson R.A. (1982). Improving Resolution in Photolithography with a Phase-Shifting Mask. IEEE Trans. Electron Devices.

[19] Banine V.Y., Koshelev K.N., Swinkels G.H.P.M. (2011). Physical Processes in EUV Sources for Microlithography. J. Phys. D Appl. Phys..

[20] Park J.S., Zhang S., She A., Chen W.T., Lin P., Yousef K.M.A., Cheng J.X., Capasso F. (2019). All-Glass, Large Metalens at Visible Wavelength Using Deep-Ultraviolet Projection Lithography. Nano Lett..

[21] Lin B.J. (1975). Deep uv lithography. J. Vac. Sci. Technol..

[22] Kitsara M., Kontziampasis D., Agbulut O., Chen Y. (2019). Heart on a Chip: Micro-Nanofabrication and Microfluidics Steering the Future of Cardiac Tissue Engineering. Microelectron. Eng..

[23] Pan D.Z., Yu P., Cho M., Ramalingam A., Kim K., Rajaram A., Shi S.X. (2008). Design for Manufacturing Meets Advanced Process Control: A Survey. J. Process Control.

[24] Chien C.F., Hsu C.Y. (2011). UNISON Analysis to Model and Reduce Step-and-Scan Overlay Errors for Semiconductor Manufacturing. J. Intell. Manuf..

[25] Saifullah M.S.M., Asbahi M., Neo D.C.J., Mahfoud Z., Tan H.R., Ha S.T., Dwivedi N., Dutta T., Bin Dolmanan S., Aabdin Z. (2022). Patterning at the Resolution Limit of Commercial Electron Beam Lithography. Nano Lett..

[26] Wang X., Dai X., Wang H., Wang J., Chen Q., Chen F., Yi Q., Tang R., Gao L., Ma L. (2023). All-Water Etching-Free Electron Beam Lithography for On-Chip Nanomaterials. ACS Nano.

[27] Lu J., Sui X., Novoselov K.S., Huang P., Xu F., Sun L. (2024). Electron Beam-Assisted Synthesis and Modification of Electrode/Separator Materials for Lithium-Ion Batteries: Progress and Prospects. Coord. Chem. Rev..

[28] Heuberger A. (1986). X-Ray Lithography. Microelectron. Eng..

[29] Bharti A., Turchet A., Marmiroli B. (2022). X-Ray Lithography for Nanofabrication: Is There a Future?. Front. Nanotechnol..

[30] Maldonado J.R., Peckerar M. (2016). X-Ray Lithography: Some History, Current Status and Future Prospects. Microelectron. Eng..

[31] Kang H., Lee D., Yang Y., Oh D.K., Seong J., Kim J., Jeon N., Kang D., Rho J. (2023). Emerging Low-Cost, Large-Scale Photonic Platforms with Soft Lithography and Self-Assembly. Photonics Insights.

[32] Wen B., Yang J., Hu C., Cai J., Zhou J. (2024). Top-Down Fabrication of Ordered Nanophotonic Structures for Biomedical Applications. Adv. Mater. Interfaces.

[33] Shimizu Y. (2021). Laser Interference Lithography for Fabrication of Planar Scale Gratings for Optical Metrology. Nanomanuf. Metrol..

[34] Liu R., Cao L., Liu D., Wang L., Saeed S., Wang Z. (2023). Laser Interference Lithography—A Method for the Fabrication of Controlled Periodic Structures. Nanomaterials.

[35] Xia Y., Whitesides G.M. (1998). Soft Lithography. Angew. Chem. Int. Ed..

[36] del Campo A., Arzt E. (2008). Fabrication Approaches for Generating Complex Micro- and Nanopatterns on Polymeric Surfaces. Chem. Rev..

[37] Chou S.Y., Krauss P.R., Renstrom P.J. (1995). Imprint of Sub-25 Nm Vias and Trenches in Polymers. Appl. Phys. Lett..

[38] Guo L.J. (2007). Nanoimprint Lithography: Methods and Material Requirements. Adv. Mater..

[39] Xu L., Wang Z., Song Q., Sun X., Liu H., Fang R., Jiang X. (2024). Microstructures Formation through Liquid-Assisted Assembly of Functional Materials for High-Performance Electronics. Adv. Funct. Mater..

[40] Wahle M., Brassat K., Ebel J., Bürger J., Lindner J.K.N., Kitzerow H.-S. (2017). Two-Dimensional Switchable Blue Phase Gratings Manufactured by Nanosphere Lithography. Opt. Express.

[41] Li J., Hu Y., Yu L., Li L., Ji D., Li L., Hu W., Fuchs H. (2021). Recent Advances of Nanospheres Lithography in Organic Electronics. Small.

[42] Ito T., Okazaki S. (2000). Pushing the Limits of Lithography. Nature.

[43] Park M., Harrison C., Chaikin P.M., Register R.A., Adamson D.H. (1997). Block Copolymer Lithography: Periodic Arrays of ~1011 Holes in 1 Square Centimeter. Science.

[44] Karayianni M., Pispas S. (2021). Block Copolymer Solution Self-Assembly: Recent Advances, Emerging Trends, and Applications. J. Polym. Sci..

[45] Gates B.D., Xu Q., Stewart M., Ryan D., Willson C.G., Whitesides G.M. (2005). New Approaches to Nanofabrication: Molding, Printing, and Other Techniques. Chem. Rev..

[46] Piner R.D., Zhu J., Xu F., Hong S., Mirkin C.A. (1999). “Dip-Pen” Nanolithography. Science.

[47] Stoykovich M.P., Kang H., Daoulas K.C., Liu G., Liu C.C., De Pablo J.J., Müller M., Nealey P.F. (2007). Directed Self-Assembly of Block Copolymers for Nanolithography: Fabrication of Isolated Features and Essential Integrated Circuit Geometries. ACS Nano.

[48] Chanda D., Shigeta K., Gupta S., Cain T., Carlson A., Mihi A., Baca A.J., Bogart G.R., Braun P., Rogers J.A. (2011). Large-Area Flexible 3D Optical Negative Index Metamaterial Formed by Nanotransfer Printing. Nat. Nanotechnol..

[49] Jeong J.W., Yang S.R., Hur Y.H., Kim S.W., Baek K.M., Yim S., Jang H.I., Park J.H., Lee S.Y., Park C.O. (2014). High-Resolution Nanotransfer Printing Applicable to Diverse Surfaces via Interface-Targeted Adhesion Switching. Nat. Commun..

[50] Liang X., Tan H., Fu Z., Chou S.Y. (2007). Air Bubble Formation and Dissolution in Dispensing Nanoimprint Lithography. Nanotechnology.

[51] van Assenbergh P., Meinders E., Geraedts J., Dodou D. (2018). Nanostructure and Microstructure Fabrication: From Desired Properties to Suitable Processes. Small.

[52] Hoffnagle J.A., Hinsberg W.D., Sanchez M., Houle F.A. (1999). Liquid Immersion Deep-Ultraviolet Interferometric Lithography. J. Vac. Sci. Technol. B.

[53] Jiang M., Liang D., Yan B., Li L., Gao M., Huang J., Lan A., Shi J. Resolution Improvement Review for the Immersion Lithography. Proceedings of the IWAPS 2022—2022 6th International Workshop on Advanced Patterning Solutions.

[54] Kazazis D., Santaclara J.G., van Schoot J., Mochi I., Ekinci Y. (2024). Extreme Ultraviolet Lithography. Nat. Rev. Methods Primers.

[55] Hasan M.W., Deeb L., Kumaniaev S., Wei C., Wang K. (2024). Recent Advances in Metal-Oxide-Based Photoresists for EUV Lithography. Micromachines.

[56] Wang X., Tao P., Wang Q., Zhao R., Liu T., Hu Y., Hu Z., Wang Y., Wang J., Tang Y. (2023). Trends in Photoresist Materials for Extreme Ultraviolet Lithography: A Review. Mater. Today.

[57] Erdmann A., Mesilhy H., Evanschitzky P. (2022). Attenuated Phase Shift Masks: A Wild Card Resolution Enhancement for Extreme Ultraviolet Lithography?. J. Micro/Nanopatterning Mater. Metrol..

[58] Xie Z., Yu W., Wang T., Zhang H., Fu Y., Liu H., Li F., Lu Z., Sun Q. (2011). Plasmonic Nanolithography: A Review. Plasmonics.

[59] Hong F., Blaikie R. (2019). Plasmonic Lithography: Recent Progress. Adv. Opt. Mater..

[60] Li X., Cui G., Xu G. (2025). The Principle and Development of Optical Maskless Lithography Based Digital Micromirror Device (DMD). Micromachines.

[61] Lin S.J., Wang W.C., Chen J.J.H., Krecinic F., Lin B.J., de Boer G., Slot E., Jager R., Steenbrink S., Kampherbeek B.-J. (2009). Imaging Performance of Production-Worthy Multiple-E-Beam Maskless Lithography. Lithogr. Asia.

[62] Klein C., Platzgummer E., Loeschner H. Projection Mask-Less Lithography and Nanopatterning with Electron and Ion Multi-Beams. Proceedings of the 26th European Mask and Lithography Conference.

[63] Tang M., Chen Z.C., Huang Z.Q., Choo Y.S., Hong M.H. (2011). Maskless Multiple-Beam Laser Lithography for Large-Area Nanostructure/Microstructure Fabrication. Appl. Opt..

[64] Schäffer E., Thurn-Albrecht T., Russell T.P., Steiner U. (2000). Electrically Induced Structure Formation and Pattern Transfer. Nature.

[65] Lee S., Jung S., Jang A.R., Hwang J., Shin H.S., Lee J., Kang D.J. (2017). An Innovative Scheme for Sub-50 Nm Patterning: Via Electrohydrodynamic Lithography. Nanoscale.

[66] Pan L., Park Y., Xiong Y., Ulin-Avila E., Wang Y., Zeng L., Xiong S., Rho J., Sun C., Bogy D.B. (2011). Maskless Plasmonic Lithography at 22 Nm Resolution. Sci. Rep..

[67] Chen Y., Xiong S. (2020). Directed Self-Assembly of Block Copolymers for Sub-10 Nm Fabrication. Int. J. Extrem. Manuf..

[68] Maekawa S., Seshimo T., Dazai T., Sato K., Hatakeyama-Sato K., Nabae Y., Hayakawa T. (2024). Chemically Tailored Block Copolymers for Highly Reliable Sub-10-Nm Patterns by Directed Self-Assembly. Nat. Commun..

[69] Haaheim J., Eby R., Nelson M., Fragala J., Rosner B., Zhang H., Athas G. (2005). Dip Pen Nanolithography (DPN): Process and Instrument Performance with NanoInk’s NSCRIPTOR System. Ultramicroscopy.

[70] Liu G., Hirtz M., Fuchs H., Zheng Z. (2019). Development of Dip-Pen Nanolithography (DPN) and Its Derivatives. Small.

[71] Chou S.Y., Krauss P.R. (1997). Imprint Lithography with Sub-10 Nm Feature Size and High Throughput. Microelectron. Eng..

[72] Basu P., Verma J., Abhinav V., Ratnesh R.K., Singla Y.K., Kumar V. (2025). Advancements in Lithography Techniques and Emerging Molecular Strategies for Nanostructure Fabrication. Int. J. Mol. Sci..

[73] Jan Z., Ahamed F., Mayer W., Patel N., Grossmann G., Stumptner M., Kuusk A. (2023). Artificial Intelligence for Industry 4.0: Systematic Review of Applications, Challenges, and Opportunities. Expert Syst. Appl..

[74] Song M.K., Kang J.H., Zhang X., Ji W., Ascoli A., Messaris I., Demirkol A.S., Dong B., Aggarwal S., Wan W. (2023). Recent Advances and Future Prospects for Memristive Materials, Devices, and Systems. ACS Nano.

[75] Zoschke K., Mackowiak P., Ngo H.D., Tschoban C., Fritsche C., Kröhnert K., Fischer T., Ndip I., Lang K.D. High-Density Flexible Substrate Technology with Thin Chip Embedding and Partial Carrier Release Option for IoT and Sensor Applications. Proceedings of the 2019 IEEE 69th Electronic Components and Technology Conference (ECTC).

[76] Choi H., Kim J., Kim W., Seong J., Park C., Choi M., Kim N., Ha J., Qiu C.W., Rho J. (2023). Realization of High Aspect Ratio Metalenses by Facile Nanoimprint Lithography Using Water-Soluble Stamps. PhotoniX.

[77] Park J.S., Lim S.W.D., Amirzhan A., Kang H., Karrfalt K., Kim D., Leger J., Urbas A., Ossiander M., Li Z. (2024). All-Glass 100 Mm Diameter Visible Metalens for Imaging the Cosmos. ACS Nano.

[78] Kazanskiy N.L., Khonina S.N., Butt M.A. (2022). Recent Development in Metasurfaces: A Focus on Sensing Applications. Nanomaterials.

[79] Wu C., Wang X., Lin L., Guo H., Wang Z.L. (2016). Paper-Based Triboelectric Nanogenerators Made of Stretchable Interlocking Kirigami Patterns. ACS Nano.

[80] Zhang X., Guo S., Han Y., Li J., Wang E. (2017). Beyond Conventional Patterns: New Electrochemical Lithography with High Precision for Patterned Film Materials and Wearable Sensors. Anal. Chem..

[81] Shneidman A.V., Becker K.P., Lukas M.A., Torgerson N., Wang C., Reshef O., Burek M.J., Paul K., McLellan J., Lončar M. (2018). All-Polymer Integrated Optical Resonators by Roll-to-Roll Nanoimprint Lithography. ACS Photonics.

[82] Oh J.Y., Lee Y., Lee T.W. (2024). Skin-Mountable Functional Electronic Materials for Bio-Integrated Devices. Adv. Healthc. Mater..

[83] Yoon G., Tanaka T., Zentgraf T., Rho J. (2021). Recent Progress on Metasurfaces: Applications and Fabrication. J. Phys. D Appl. Phys..

[84] Ali A., Mitra A., Aïssa B. (2022). Metamaterials and Metasurfaces: A Review from the Perspectives of Materials, Mechanisms and Advanced Metadevices. Nanomaterials.

[85] Chen Y., Lu Z., Wang X., Cui Z., Pan G., Zhou Y., Muñoz M., Hao C., Yonghua L., Garcia N. (2007). Fabrication of Ferromagnetic Nanoconstrictions by Electron Beam Lithography Using LOR/PMMA Bilayer Technique. Microelectron. Eng..

[86] Cui B., Veres T. (2008). High Resolution Electron Beam Lithography of PMGI Using Solvent Developers. Microelectron. Eng..

[87] Chen Y., Schwanecke A.S., Fedotov V.A., Khardikov V.V., Mladyonov P.L., Prosvirnin S.L., Rogacheva A.V., Zheludev N.I., Huq E. (2009). Electron Beam Lithography for High Density Meta Fish Scale Operational at Optical Frequency. Microelectron. Eng..

[88] Bonam R., Verhagen P., Munder A., Hartley J. (2010). Performance Characterization of Negative Resists for Sub-10-Nm Electron Beam Lithography. J. Vac. Sci. Technol. B.

[89] Choi S., Jin N., Kumar V., Adesida I., Shannon M. (2007). Effects of Developer Temperature on Electron-Beam-Exposed Hydrogen Silsesquioxane Resist for Ultradense Silicon Nanowire Fabrication. J. Vac. Sci. Technol. B.

[90] Lanniel M., Lu B., Chen Y., Allen S., Buttery L., Williams P., Huq E., Alexander M. (2011). Patterning the Mechanical Properties of Hydrogen Silsesquioxane Films Using Electron Beam Irradiation for Application in Mechano Cell Guidance. Thin Solid Film..

[91] Mao Q., Zhu J., Wang Z. (2025). Quantitative Evaluation of Residual Resist in Electron Beam Lithography Based on Scanning Electron Microscopy Imaging and Thresholding Segmentation Algorithm. Nanotechnology.

[92] Grigorescu A.E., Hagen C.W. (2009). Resists for Sub-20-Nm Electron Beam Lithography with a Focus on HSQ: State of the Art. Nanotechnology.

[93] Kolodziej C.M., Maynard H.D. (2012). Electron-Beam Lithography for Patterning Biomolecules at the Micron and Nanometer Scale. Chem. Mater..

[94] Okazaki S. (2015). High Resolution Optical Lithography or High Throughput Electron Beam Lithography: The Technical Struggle from the Micro to the Nano-Fabrication Evolution. Microelectron. Eng..

[95] Chen Y. (2015). Nanofabrication by Electron Beam Lithography and Its Applications: A Review. Microelectron. Eng..

[96] Zhu C., Ekinci H., Pan A., Cui B., Zhu X. (2024). Electron Beam Lithography on Nonplanar and Irregular Surfaces. Microsyst. Nanoeng..

[97] Sin Tan Y., Wang H., Wang H., Pan C., Yang J.K.W. (2023). High-Throughput Fabrication of Large-Scale Metasurfaces Using Electron-Beam Lithography with SU-8 Gratings for Multilevel Security Printing. Photonics Res..

[98] Zhao D., Chang B., Beleggia M. (2020). Electron-Beam Patterning of Vapor-Deposited Solid Anisole. ACS Appl. Mater. Interfaces.

[99] Maoz R., Berson J., Burshtain D., Nelson P., Zinger A., Bitton O., Sagiv J. (2018). Interfacial Electron Beam Lithography: Chemical Monolayer Nanopatterning via Electron-Beam-Induced Interfacial Solid-Phase Oxidation. ACS Nano.

[100] Rommel M., Nilsson B., Jedrasik P., Bonanni V., Dmitriev A., Weis J. (2013). Sub-10 Nm Resolution after Lift-off Using HSQ/PMMA Double Layer Resist. Microelectron. Eng..

[101] Bourdillon A.J., Boothroyd C.B., Kong J.R. (1999). Demagnification in Proximity X-RAY Lithography. J. Phys. D Appl. Phys.

[102] Malek C.K., Yajamanyam S. (2000). Evaluation of Alternative Development Process for High-Aspect-Ratio Poly(Methylmethacrylate) Microstructures in Deep x-Ray Lithography. J. Vac. Sci. Technol. B.

[103] Hirai Y., Hafizovic S., Matsuzuka N., Korvink J.G., Tabata O. (2006). Validation of X-Ray Lithography and Development Simulation System for Moving Mask Deep X-Ray Lithography. J. Microelectromechanical Syst..

[104] Falcaro P., Malfatti L., Vaccari L., Amenitsch H., Marmiroli B., Grenci G., Innocenzi P. (2009). Fabrication of Advanced Functional Devices Combining Soft Chemistry with X-Ray Lithography in One Step. Adv. Mater..

[105] Costacurta S., Malfatti L., Patelli A., Falcaro P., Amenitsch H., Marmiroli B., Grenci G., Piccinini M., Innocenzi P. (2010). Deep X-Ray Lithography for Direct Patterning of PECVD Films. Plasma Process. Polym..

[106] Faustini M., Marmiroli B., Malfatti L., Louis B., Krins N., Falcaro P., Grenci G., Laberty-Robert C., Amenitsch H., Innocenzi P. (2011). Direct Nano-in-Micropatterning of TiO2 Thin Layers and TiO 2/Pt Nanoelectrode Arrays by Deep X-Ray Lithography. J. Mater. Chem..

[107] Späth A., Tu F., Vollnhals F., Drost M., Krick Calderón S., Watts B., Fink R.H., Marbach H. (2016). Additive Fabrication of Nanostructures with Focused Soft X-Rays. RSC Adv..

[108] Ceddia D., Aminzadeh A., Cook P.K., Pelliccia D., Kingston A.M., Paganin D.M. (2023). Universal Mask for Hard x Rays. Optica.

[109] Li T., Kahnt M., Sheppard T.L., Yang R., Falch K.V., Zvagelsky R., Villanueva-Perez P., Wegener M., Lyubomirskiy M. (2024). X-Ray Multibeam Ptychography at up to 20 KeV: Nano-Lithography Enhances X-Ray Nano-Imaging. Adv. Sci..

[110] Cerrina F. (2000). X-Ray Imaging: Applications to Patterning and Lithography. J. Phys. D Appl. Phys.

[111] Macintyre D., Thoms S. (1997). High Resolution Electron Beam Lithography Studies on Shipley Chemically Amplified DUV Resists. Microelectron. Eng..

[112] Chen Y., Macintyre D., Thoms S. (1999). Electron Beam Lithography Process for T- and Γ-Shaped Gate Fabrication Using Chemically Amplified DUV Resists and PMMA. J. Vac. Sci. Technol. B.

[113] Chen Y., Peng K., Cui Z. (2004). Fabrication of Ultra-Short T Gates by a Two-Step Electron Beam Lithography Process. Microelectron. Eng..

[114] Park S.-J., Liddle J.A., Persaud A., Allen F.I., Schenkel T., Bokor J. (2004). Formation of 15nm Scale Coulomb Blockade Structures in Silicon by Electron Beam Lithography with a Bilayer Resist Process. J. Vac. Sci. Technol. B.

[115] Hu W., Sarveswaran K., Lieberman M., Bernstein G.H. (2004). Sub-10 Nm Electron Beam Lithography Using Cold Development of Poly(Methylmethacrylate). J. Vac. Sci. Technol. B.

[116] Zhang W., Potts A., Bagnall D.M., Davidson B.R. (2006). Large Area All-Dielectric Planar Chiral Metamaterials by Electron Beam Lithography. J. Vac. Sci. Technol. B.

[117] Kim S.J., Hwang J.S., Park J.E., Yang M., Kim S. (2021). Exploring SERS from Complex Patterns Fabricated by Multi-Exposure Laser Interference Lithography. Nanotechnology.

[118] Brueck S.R.J. (2005). Optical and Interferometric Lithography -Nanotechnology Enablers. Proc. IEEE.

[119] Xie Q., Hong M.H., Tan H.L., Chen G.X., Shi L.P., Chong T.C. (2008). Fabrication of Nanostructures with Laser Interference Lithography. J. Alloys Compd..

[120] De Boor J., Geyer N., Gösele U., Schmidt V. (2009). Three-Beam Interference Lithography: Upgrading a Lloyd’s Interferometer for Single-Exposure Hexagonal Patterning. Opt. Lett..

[121] Vala M., Homola J. (2014). Flexible Method Based on Four-Beam Interference Lithography for Fabrication of Large Areas of Perfectly Periodic Plasmonic Arrays. Opt. Express.

[122] Ren Y., Wang X., Di X., Jia T., Chen T., Zhang L., Yang H., Qi Y., Tang C. (2023). Theoretical Study on Fabrication of Sub-Wavelength Structures via Combining Low-Order Guided Mode Interference Lithography with Sample Rotation. J. Opt..

[123] Luo L., Shan S., Li X. (2024). A Review: Laser Interference Lithography for Diffraction Gratings and Their Applications in Encoders and Spectrometers. Sensors.

[124] Chua J.K., Murukeshan V.M. (2009). Patterning of Two-Dimensional Nanoscale Features Using Gratingbased Multiple Beams Interference Lithography. Phys. Scr..

[125] Ahn J., Hong S., Shim Y.S., Park J. (2020). Electroplated Functional Materials with 3d Nanostructures Defined by Advanced Optical Lithography and Their Emerging Applications. Appl. Sci..

[126] He M., Zhang Z., Cao C., Zhou G., Kuang C., Liu X. (2022). 3D Sub-Diffraction Printing by Multicolor Photoinhibition Lithography: From Optics to Chemistry. Laser Photon. Rev..

[127] Capraro G., Lipkin M., Möller M., Bolten J., Lemme M.C. (2023). Phase Mask Pinholes as Spatial Filters for Laser Interference Lithography. Adv. Photonics Res..

[128] Xue D., Deng X., Dun X., Wang J., Wang Z., Cheng X. (2024). Improving Grating Duty Cycle Uniformity: Amplitude-Splitting Flat-Top Beam Laser Interference Lithography. Appl. Opt..

[129] Xue G., Lu H., Li X., Zhou Q., Wu G., Wang X., Zhai Q., Ni K. (2020). Patterning Nanoscale Crossed Grating with High Uniformity by Using Two-Axis Lloyd’s Mirrors Based Interference Lithography. Opt. Express.

[130] Hong F., Blaikie R. (2019). Reflective Metamaterial Polarizer Enabled by Solid-Immersion Lloyd’s Mirror Interference Lithography. J. Vac. Sci. Technol. B.

[131] Perl A., Reinhoudt D.N., Huskens J. (2009). Microcontact Printing: Limitations and Achievements. Adv. Mater..

[132] Zemła J., Szydlak R., Gajos K., Kozłowski Ł., Zieliński T., Luty M., Øvreeide I.H., Prot V.E., Stokke B.T., Lekka M. (2023). Plasma Treatment of PDMS for Microcontact Printing (ΜCP) of Lectins Decreases Silicone Transfer and Increases the Adhesion of Bladder Cancer Cells. ACS Appl. Mater. Interfaces.

[133] Sun M., Zhang J., Xuanyuan T., Liu X., Liu W. (2024). Facile and Rapid Microcontact Printing of Additive-Free Polydimethylsiloxane for Biological Patterning Diversity. ACS Appl. Mater. Interfaces.

[134] Ghosh A., Bandyopadhyay D., Sharma A. (2018). Electric Field Mediated Elastic Contact Lithography of Thin Viscoelastic Films for Miniaturized and Multiscale Patterns. Soft Matter.

[135] Cordero-Guerrero J., Jiménez-Thuel G., Paniagua S.A. (2023). Sub-Micron Patterning of Metal Oxide Surfaces via Microcontact Printing and Microtransfer Molding of Amphiphilic Molecules and Antifouling Application. J. Mater. Res..

[136] Unno N., Mäkelä T. (2023). Thermal Nanoimprint Lithography—A Review of the Process, Mold Fabrication, and Material. Nanomaterials.

[137] Sun Y.L., Jevasuwan W., Fukata N. (2024). Top-down Fabrication of Si Nanotube Arrays Using Nanoimprint Lithography and Spacer Patterning for Electronic and Optoelectronic Applications. Mater. Today Nano.

[138] Modaresialam M., Chehadi Z., Bottein T., Abbarchi M., Grosso D. (2021). Nanoimprint Lithography Processing of Inorganic-Based Materials. Chem. Mater..

[139] Cox L.M., Martinez A.M., Blevins A.K., Sowan N., Ding Y., Bowman C.N. (2020). Nanoimprint Lithography: Emergent Materials and Methods of Actuation. Nano Today.

[140] Jeon S., Park R., Jeong J., Heo G., Lee J., Shin M.C., Kwon Y.W., Yang J.C., Park W.I., Kim K.S. (2021). Rotating Cylinder-Assisted Nanoimprint Lithography for Enhanced Chemisorbable Filtration Complemented by Molecularly Imprinted Polymers. Small.

[141] McGrath F., Qian J., Gwynne K., Kumah C., Daly D., Hrelescu C., Zhang X., O’Carroll D.M., Louise Bradley A. (2021). Structural, Optical, and Electrical Properties of Silver Gratings Prepared by Nanoimprint Lithography of Nanoparticle Ink. Appl. Surf. Sci..

[142] Cai J., Li G., Zhou J., Li W. (2023). Di Hydrodynamics and Solid Mechanics Structural Analysis of Mold Deformation in Nanoimprint Lithography. Extrem. Mech. Lett..

[143] Mao H., Zhang L., Wen L., Huang L., Tan L., Chen Y. (2023). Nanoimprint Lithography-Dependent Vertical Composition Gradient in Pseudo-Planar Heterojunction Organic Solar Cells Combined with Sequential Deposition. Adv. Funct. Mater..

[144] Kim K.H., Huh Y., Song I., Ryu D.Y., Son J.G., Bang J. (2024). Recent Advances in Block Copolymer Self-Assembly: From the Lowest to the Highest. J. Polym. Sci..

[145] Cheng J.Y., Ross C.A., Thomas E.L., Smith H.I., Vancso G.J. (2002). Fabrication of Nanostructures with Long-Range Order Using Block Copolymer Lithography. Appl. Phys. Lett..

[146] Park S.M., Stoykovich M.P., Ruiz R., Zhang Y., Black C.T., Nealey P.F. (2007). Directed Assembly of Lamellae-Forming Block Copolymers by Using Chemically and Topographically Patterned Substrates. Adv. Mater..

[147] Jung Y.S., Jung W.C., Tuller H.L., Ross C.A. (2008). Nanowire Conductive Polymer Gas Sensor Patterned Using Self-Assembled Block Copolymer Lithography. Nano Lett..

[148] Shin D.O., Jeong J.R., Han T.H., Koo C.M., Park H.J., Lim Y.T., Kim S.O. (2010). A Plasmonic Biosensor Array by Block Copolymer Lithography. J. Mater. Chem..

[149] Nunes S.P., Behzad A.R., Hooghan B., Sougrat R., Karunakaran M., Pradeep N., Vainio U., Peinemann K.V. (2011). Switchable PH-Responsive Polymeric Membranes Prepared via Block Copolymer Micelle Assembly. ACS Nano.

[150] Jeong C.K., Jin H.M., Ahn J.H., Park T.J., Yoo H.G., Koo M., Choi Y.K., Kim S.O., Lee K.J. (2014). Electrical Biomolecule Detection Using Nanopatterned Silicon via Block Copolymer Lithography. Small.

[151] Nunns A., Gwyther J., Manners I. (2013). Inorganic Block Copolymer Lithography. Polymer.

[152] Cummins C., Lundy R., Walsh J.J., Ponsinet V., Fleury G., Morris M.A. (2020). Enabling Future Nanomanufacturing through Block Copolymer Self-Assembly: A Review. Nano Today.

[153] Kulkarni A.A., Doerk G.S. (2022). Thin Film Block Copolymer Self-Assembly for Nanophotonics. Nanotechnology.

[154] Pinto-Gómez C., Pérez-Murano F., Bausells J., Villanueva L.G., Fernández-Regúlez M. (2020). Directed Self-Assembly of Block Copolymers for the Fabrication of Functional Devices. Polymers.

[155] Mokarian-Tabari P., Senthamaraikannan R., Glynn C., Collins T.W., Cummins C., Nugent D., O’Dwyer C., Morris M.A. (2017). Large Block Copolymer Self-Assembly for Fabrication of Subwavelength Nanostructures for Applications in Optics. Nano Lett..

[156] Jin H.M., Kim J.Y., Heo M., Jeong S.J., Kim B.H., Cha S.K., Han K.H., Kim J.H., Yang G.G., Shin J. (2018). Ultralarge Area Sub-10 Nm Plasmonic Nanogap Array by Block Copolymer Self-Assembly for Reliable High-Sensitivity SERS. ACS Appl. Mater. Interfaces.

[157] Fei H.F., Yavitt B.M., Hu X., Kopanati G., Ribbe A., Watkins J.J. (2019). Influence of Molecular Architecture and Chain Flexibility on the Phase Map of Polystyrene-Block-Poly(Dimethylsiloxane) Brush Block Copolymers. Macromolecules.

[158] Huang H., Liu R., Ross C.A., Alexander-Katz A. (2020). Self-Directed Self-Assembly of 3D Tailored Block Copolymer Nanostructures. ACS Nano.

[159] Sun Z., Liu R., Su T., Huang H., Kawamoto K., Liang R., Liu B., Zhong M., Alexander-Katz A., Ross C.A. (2023). Emergence of Layered Nanoscale Mesh Networks through Intrinsic Molecular Confinement Self-Assembly. Nat. Nanotechnol..

[160] Putranto A.F., Petit-Etienne C., Cavalaglio S., Cabannes-Boué B., Panabiere M., Forcina G., Fleury G., Kogelschatz M., Zelsmann M. (2024). Controlled Anisotropic Wetting by Plasma Treatment for Directed Self-Assembly of High-χ Block Copolymers. ACS Appl. Mater. Interfaces.

[161] Ndaya D., Bosire R., Kasi R.M. (2020). Spherical Photonic Nanostructures from High Molecular Weight Liquid Crystalline Brushlike Block Copolymers. ACS Appl. Polym. Mater..

[162] Kim J.H., Jin H.M., Yang G.G., Han K.H., Yun T., Shin J.Y., Jeong S.J., Kim S.O. (2020). Smart Nanostructured Materials Based on Self-Assembly of Block Copolymers. Adv. Funct. Mater..

[163] Yang G.G., Choi H.J., Han K.H., Kim J.H., Lee C.W., Jung E.I., Jin H.M., Kim S.O. (2022). Block Copolymer Nanopatterning for Nonsemiconductor Device Applications. ACS Appl. Mater. Interfaces.

[164] Telecka A., Li T., Ndoni S., Taboryski R. (2018). Nanotextured Si Surfaces Derived from Block-Copolymer Self-Assembly with Superhydrophobic, Superhydrophilic, or Superamphiphobic Properties. RSC Adv..

[165] Song C., Ye B., Xu J., Chen J., Shi W., Yu C., An C., Zhu J., Zhang W. (2022). Large-Area Nanosphere Self-Assembly Monolayers for Periodic Surface Nanostructures with Ultrasensitive and Spatially Uniform SERS Sensing. Small.

[166] Chen X., Cui A., He M., Yan M., Zhang X., Ruan J., Yang S. (2023). Slippery Au Nanosphere Monolayers with Analyte Enrichment and SERS Enhancement Functions. Nano Lett..

[167] Lu X., Huang H., Wang X., Li X., Liu G., Chai L., Zhou L., Shao J. (2022). Quasi-Ordered Nanosphere-Based Photonic Crystals with High-Fastness Structural Colors via Screen Printing: Implications for Textile Printing and Dyeing. ACS Appl. Nano Mater..

[168] Wu Y., Nan J., Ren J., Meng Z., Zhang S., Wu S. (2022). Polarization-Dependent Structural Colors in ZnS Nanosphere-Based Photonic Crystals for Anticounterfeiting Applications. ACS Appl. Nano Mater..

[169] Schletz D., Schultz J., Potapov P.L., Steiner A.M., Krehl J., König T.A.F., Mayer M., Lubk A., Fery A. (2021). Exploiting Combinatorics to Investigate Plasmonic Properties in Heterogeneous Ag-Au Nanosphere Chain Assemblies. Adv. Opt. Mater..

[170] Xu K., Chen J. (2020). High-Resolution Scanning Probe Lithography Technology: A Review. Appl. Nanosci..

[171] Agarwal G., Naik R.R., Stone M.O. (2003). Immobilization of Histidine-Tagged Proteins on Nickel by Electrochemical Dip Pen Nanolithography. J. Am. Chem. Soc..

[172] Salaita K., Lee S.W., Wang X., Huang L., Dellinger T.M., Liu C., Mirkin C.A. (2005). Sub-100 Nm, Centimeter-Scale, Parallel Dip-Pen Nanolithography. Small.

[173] Mirkin C.A. (2007). The Power of the Pen: Development of Parallel Dip-Pen Nanolithography. ACS Nano.

[174] Sekula S., Fuchs J., Weg-Remers S., Nagel P., Schuppler S., Fragala J., Theilacker N., Franzreb M., Wingren C., Ellmark P. (2008). Multiplexed Lipid Dip-Pen Nanolithography on Subcellular Scales for the Templating of Functional Proteins and Cell Culture. Small.

[175] Liu X., Li Y., Zheng Z. (2010). Programming Nanostructures of Polymer Brushes by Dip-Pen Nanodisplacement Lithography (DNL). Nanoscale.

[176] Schlichter L., Bosse F., Tyler B.J., Arlinghaus H.F., Ravoo B.J. (2023). Patterning of Hydrophilic and Hydrophobic Gold and Magnetite Nanoparticles by Dip Pen Nanolithography. Small.

[177] Weeks B.L., Noy A., Miller A.E., De Yoreo J.J. (2002). Effect of Dissolution Kinetics on Feature Size in Dip-Pen Nanolithography. Phys. Rev. Lett..

[178] Zhang H., Elghanian R., Amro N.A., Disawal S., Eby R. (2004). Dip Pen Nanolithography Stamp Tip. Nano Lett..

[179] Nelson B.A., King W.P., Laracuente A.R., Sheehan P.E., Whitman L.J. (2006). Direct Deposition of Continuous Metal Nanostructures by Thermal Dip-Pen Nanolithography. Appl. Phys. Lett..

[180] Wang Y., Giam L.R., Park M., Lenhert S., Fuchs H., Mirkin C.A. (2008). A Self-Correcting Inking Strategy for Cantilever Arrays Addressed by an Inkjet Printer and Used for Dip-Pen Nanolithography. Small.

[181] Lee W.K., Whitman L.J., Lee J., King W.P., Sheehan P.E. (2008). The Nanopatterning of a Stimulus-Responsive Polymer by Thermal Dip-Pen Nanolithography. Soft Matter.

[182] Chai J., Huo F., Zheng Z., Giam L.R., Shim W., Mirkin C.A. (2010). Scanning Probe Block Copolymer Lithography. Proc. Natl. Acad. Sci. USA.

[183] Wu C.C., Reinhoudt D.N., Otto C., Subramaniam V., Velders A.H. (2011). Strategies for Patterning Biomolecules with Dip-Pen Nanolithography. Small.

[184] Hirtz M., Oikonomou A., Georgiou T., Fuchs H., Vijayaraghavan A. (2013). Multiplexed Biomimetic Lipid Membranes on Graphene by Dip-Pen Nanolithography. Nat. Commun..

[185] Gavutis M., Navikas V., Rakickas T., Vaitekonis Š., Valiokas R. (2016). Lipid Dip-Pen Nanolithography on Self-Assembled Monolayers. J. Micromechan. Microengin..

[186] Shin H.W., Son J.Y. (2018). Ferromagnetic Fe2O3 Nanopatterns Prepared Using Dip-Pen Lithography. Solid State Commun..

[187] Liu H.Y., Kumar R., Takai M., Hirtz M. (2020). Enhanced Stability of Lipid Structures by Dip-Pen Nanolithography on Block-Type MPC Copolymer. Molecules.

[188] Saygin V., Xu B., Andersson S.B., Brown K.A. (2021). Closed-Loop Nanopatterning of Liquids with Dip-Pen Nanolithography. ACS Appl. Mater. Interfaces.

[189] Yadav K.K., Shamir D., Kornweitz H., Peled Y., Zohar M., Burg A. (2024). Development of Meta-Chemical Surface by Dip-Pen Nanolithography for Precise Electrochemical Lead Sensing. Small Methods.

[190] Dawood F., Wang J., Schulze P.A., Sheehan C.J., Buck M.R., Dennis A.M., Majumder S., Krishnamurthy S., Ticknor M., Staude I. (2018). The Role of Liquid Ink Transport in the Direct Placement of Quantum Dot Emitters onto Sub-Micrometer Antennas by Dip-Pen Nanolithography. Small.

[191] Li H., Wang Z., Huo F., Wang S. (2021). Dip-Pen Nanolithography(DPN): From Micro/Nano-Patterns to Biosensing. Chem. Res. Chin. Univ..

[192] Liu G., Petrosko S.H., Zheng Z., Mirkin C.A. (2020). Evolution of Dip-Pen Nanolithography (DPN): From Molecular Patterning to Materials Discovery. Chem. Rev..

[193] Okbi R., Alkrenawi M., Yadav K.K., Shamir D., Kornweitz H., Peled Y., Zohar M., Burg A. (2024). Dip-Pen Nanolithography-Based Fabrication of Meta-Chemical Surface for Heavy Metal Detection: Role of Poly-Methyl Methacrylate in Sensor Sensitivity. Small Sci..

[194] Yang S.N., Liu X.Q., Zheng J.X., Lu Y.M., Gao B.R. (2020). Periodic Microstructures Fabricated by Laser Interference with Subsequent Etching. Nanomaterials.

[195] Baquedano E., Martinez R.V., Llorens J.M., Postigo P.A. (2017). Fabrication of Silicon Nanobelts and Nanopillars by Soft Lithography for Hydrophobic and Hydrophilic Photonic Surfaces. Nanomaterials.

[196] Qiu M., Du W., Zhou S., Cai P., Luo Y., Wang X., Yang R., Zhao J. (2023). Recent Progress in Non-Photolithographic Patterning of Polymer Thin Films. Prog. Polym. Sci..

[197] Miranda I., Souza A., Sousa P., Ribeiro J., Castanheira E.M.S., Lima R., Minas G. (2022). Properties and Applications of PDMS for Biomedical Engineering: A Review. J. Funct. Biomater..

[198] Thanner C., Eibelhuber M. (2021). UV Nanoimprint Lithography: Geometrical Impact on Filling Properties of Nanoscale Patterns. Nanomaterials.

[199] Rimböck J., Schuster P., Vsetecka L., Thanner C. (2024). UV Nanoimprint Lithography—Impact of Coating Techniques on Pattern Quality. Nanomanufacturing.

[200] Chu Z., Xue C., Shao K., Xiang L., Zhao X., Chen C., Pan J., Lin D. (2022). Photonic Crystal-Embedded Molecularly Imprinted Contact Lenses for Controlled Drug Release. ACS Appl. Bio Mater..

[201] Menshikov E., Lazarenko P., Kovalyuk V., Dubkov S., Maslova N., Prokhodtsov A., Vorobyov A., Kozyukhin S., Goltsman G., Sinev I.S. (2024). Reversible Laser Imprinting of Phase Change Photonic Structures in Integrated Waveguides. ACS Appl. Mater. Interfaces.

[202] Muehlberger M. (2022). Nanoimprinting of Biomimetic Nanostructures. Nanomanufacturing.

[203] Nowduri B., Schulte S., Decker D., Schäfer K.H., Saumer M. (2020). Biomimetic Nanostructures Fabricated by Nanoimprint Lithography for Improved Cell-Coupling. Adv. Funct. Mater..

[204] Oopath S.V., Baji A., Abtahi M. (2022). Biomimetic Rose Petal Structures Obtained Using UV-Nanoimprint Lithography. Polymers.

[205] Ahn J., Kwon S., Jung S., Lee W.S., Jeong J., Lim H., Shin Y.B., Lee J.J. (2018). Fabrication of Pyrrole-Based Electrochemical Biosensor Platform Using Nanoimprint Lithography. Adv. Mater. Interfaces.

[206] Lee S.W., Lee K.S., Ahn J., Lee J.J., Kim M.G., Shin Y.B. (2011). Highly Sensitive Biosensing Using Arrays of Plasmonic Au Nanodisks Realized by Nanoimprint Lithography. ACS Nano.

[207] Zanut A., Cian A., Cefarin N., Pozzato A., Tormen M. (2020). Nanoelectrode Arrays Fabricated by Thermal Nanoimprint Lithography for Biosensing Application. Biosensors.

[208] Fialkova S., Yarmolenko S., Krishnaswamy A., Sankar J., Shanov V., Schulz M.J., Desai S. (2024). Nanoimprint Lithography for Next-Generation Carbon Nanotube-Based Devices. Nanomaterials.

[209] Michalska M., Laney S.K., Li T., Portnoi M., Mordan N., Allan E., Tiwari M.K., Parkin I.P., Papakonstantinou I. (2021). Bioinspired Multifunctional Glass Surfaces through Regenerative Secondary Mask Lithography. Adv. Mater..

[210] Chau Y.F.C., Chen K.H., Chiang H.P., Lim C.M., Huang H.J., Lai C.H., Kumara N.T.R.N. (2019). Fabrication and Characterization of a Metallic–Dielectric Nanorod Array by Nanosphere Lithography for Plasmonic Sensing Application. Nanomaterials.

[211] Domonkos M., Kromka A. (2022). Nanosphere Lithography-Based Fabrication of Spherical Nanostructures and Verification of Their Hexagonal Symmetries by Image Analysis. Symmetry.

[212] Sanders D.P. (2010). Advances in Patterning Materials for 193 Nm Immersion Lithography. Chem. Rev..

[213] Song J., Kim C.H., Lee G.W. (2022). A Study on the Resolution and Depth of Focus of ArF Immersion Photolithography. Micromachines.

[214] Vakarin V., Melati D., Dinh T.T.D., Le Roux X., Kan W.K.K., Dupré C., Szelag B., Monfray S., Boeuf F., Cheben P. (2021). Metamaterial-Engineered Silicon Beam Splitter Fabricated with Deep Uv Immersion Lithography. Nanomaterials.

[215] Li T., Liu Y., Sun Y., Yan X., Wei P., Li Y. (2020). Vectorial Pupil Optimization to Compensate Polarization Distortion in Immersion Lithography System. Opt. Express.

[216] Khaidarov E., Eschimese D., Lai K.H., Huang A., Fu Y.H., Lin Q., Paniagua-Dominguez R., Kuznetsov A.I. (2022). Large-Scale Vivid Metasurface Color Printing Using Advanced 12-in. Immersion Photolithography. Sci. Rep..

[217] Song J., Oh J.S., Bak M.J., Kang I.S., Lee S.J., Lee G.W. (2022). 300 Mm Large Area Wire Grid Polarizers with 50 Nm Half-Pitch by ArF Immersion Lithography. Nanomaterials.

[218] Lim G., Lee K., Choi S., Yoon H.J. (2023). Organometallic and Coordinative Photoresist Materials for EUV Lithography and Related Photolytic Mechanisms. Coord. Chem. Rev..

[219] Lodha J.K., Pollentier I., Conard T., Vallat R., De Gendt S., Armini S. (2022). Self-Assembled Monolayers as Inhibitors for Area-Selective Deposition: A Novel Approach towards Resist-Less EUV Lithography. Appl. Surf. Sci..

[220] Wang D., Xu R., Zhou D., Zhao J., Zhang J., Chen P., Peng X. (2024). Zn-Ti Oxo Cluster Photoresists for EUV Lithography: Cluster Structure and Lithographic Performance. Chem. Eng. J..

[221] Choi H.W., Nam K.B., Shin D.W. (2023). Graphite Pellicle: Physical Shield for Next-Generation EUV Lithography Technology. Adv. Mater. Interfaces.

[222] Kim K., Lee J.W., Park B.G., Oh H.T., Ku Y., Lee J.K., Lim G., Lee S. (2022). Investigation of Correlative Parameters to Evaluate EUV Lithographic Performance of PMMA. RSC Adv..

[223] Wagner C., Harned N. (2010). EUV Lithography: Lithography Gets Extreme. Nat. Photonics.

[224] Park Y., Song S.W., Hong J., Jang H., Lee G.R., Kim G.Y., Jung Y.S. (2024). Si-Containing Reverse-Gradient Block Copolymer for Inorganic Pattern Amplification in EUV Lithography. ACS Macro Lett..

[225] Tseng L.-T., Karadan P., Kazazis D., Constantinou C., Stock T.J.Z., Curson N.J., Schofield S.R., Muntwiler M., Aeppli G., Ekinci Y. (2023). Resistless EUV Lithography: Photon-Induced Oxide Patterning on Silicon. Sci. Adv..

[226] Zhang Z., Li S., Wang X., Cheng W. (2021). Fast Heuristic-Based Source Mask Optimization for EUV Lithography Using Dual Edge Evolution and Partial Sampling. Opt. Express.

[227] Wu R., Dong L., Ma X., Wei Y. (2021). Compensation of EUV Lithography Mask Blank Defect Based on an Advanced Genetic Algorithm. Opt. Express.

[228] Wang J., Su X., Su Y., Wei Y. (2024). Probability Distribution-Based Method for Aberration Budgeting in EUV Lithography. Opt. Express.

[229] Jiang B., Feng C., Li C., Bai Z., Wan W., Xiang D., Gu Q., Wang K., Zhang Q., Huang D. (2022). A Synchrotron-Based Kilowatt-Level Radiation Source for EUV Lithography. Sci. Rep..

[230] Liu J., Liu J., Deng Q., Feng J., Zhou S., Hu S. (2020). Intensity Modulation Based Optical Proximity Optimization for the Maskless Lithography. Opt. Express.

[231] Du J., Jiang J., Sun S., Li F., Yu S., Chen Q., Yang F., Yan W. (2024). Simple Visual Focusing and Alignment Technology for Digital Lithography. Opt. Lasers Eng..

[232] Chien H.L., Chiu Y.H., Lee Y.C. (2021). Maskless Lithography Based on Oblique Scanning of Point Array with Digital Distortion Correction. Opt. Lasers Eng..

[233] Huang S.Z., Li M.J., Wang L., Su Y.S., Wang F.T. (2020). Research on Maskless Lithography System Based on Digital Oblique Scanning Strategy. J. Laser Micro Nanoeng..

[234] Silver C.S., Spallas J.P., Muray L.P. (2007). Multiple Beam Sub-80-Nm Lithography with Miniature Electron Beam Column Arrays. J. Vac. Sci. Technol. B.

[235] Groves T.R., Kendall R.A. (1998). Distributed, Multiple Variable Shaped Electron Beam Column for High Throughput Maskless Lithography. J. Vac. Sci. Technol. B.

[236] Jang J., Kim Y., Kim S., Jung H., Hahn J.W. (2011). Design of a Contact Probe with High Positioning Accuracy for Plasmonic Lithography. Scanning.

[237] Kim S., Jung H., Kim Y., Jang J., Hahn J.W. (2012). Resolution Limit in Plasmonic Lithography for Practical Applications beyond 2x-Nm Half Pitch. Adv. Mater..

[238] Dong J., Liu J., Kang G., Xie J., Wang Y. (2014). Pushing the Resolution of Photolithography down to 15nm by Surface Plasmon Interference. Sci. Rep..

[239] Gao P., Yao N., Wang C., Zhao Z., Luo Y., Wang Y., Gao G., Liu K., Zhao C., Luo X. (2015). Enhancing Aspect Profile of Half-Pitch 32 Nm and 22 Nm Lithography with Plasmonic Cavity Lens. Appl. Phys. Lett..

[240] Chen X., Yang F., Zhang C., Zhou J., Guo L.J. (2016). Large-Area High Aspect Ratio Plasmonic Interference Lithography Utilizing a Single High-k Mode. ACS Nano.

[241] Han D., Park C., Jung H., Hahn J.W. (2016). Calibration of Exposure Dose for Nanoscale Plasmonic Lithography with Microsized Far-Field Spot Patterns. J. Micromechan. Microengin..

[242] Chen X., Zhang C., Yang F., Liang G., Li Q., Guo L.J. (2017). Plasmonic Lithography Utilizing Epsilon Near Zero Hyperbolic Metamaterial. ACS Nano.

[243] Liu L., Zhang X., Zhao Z., Pu M., Gao P., Luo Y., Jin J., Wang C., Luo X. (2017). Batch Fabrication of Metasurface Holograms Enabled by Plasmonic Cavity Lithography. Adv. Opt. Mater..

[244] Jung H., Park C., Oh S., Hahn J.W. (2017). Nanoscale 2.5-Dimensional Surface Patterning with Plasmonic Lithography. Sci. Rep..

[245] Gao P., Li X., Zhao Z., Ma X., Pu M., Wang C., Luo X. (2017). Pushing the Plasmonic Imaging Nanolithography to Nano-Manufacturing. Opt. Commun..

[246] Ueno K., Takabatake S., Nishijima Y., Mizeikis V., Yokota Y., Misawa H. (2010). Nanogap-Assisted Surface Plasmon Nanolithography. J. Phys. Chem. Lett..

[247] Liang G., Chen X., Zhao Q., Guo L.J. (2018). Achieving Pattern Uniformity in Plasmonic Lithography by Spatial Frequency Selection. Nanophotonics.

[248] Huang J., Xu K., Hu J., Yuan D., Li J., Qiao J., Xu S. (2022). Self-Aligned Plasmonic Lithography for Maskless Fabrication of Large-Area Long-Range Ordered 2D Nanostructures. Nano Lett..

[249] Ding H., Liu L., Li Z., Dong L., Wei Y., Ye T. (2023). Plasmonic Lithography Fast Imaging Model Based on the Decomposition Machine Learning Method. Opt. Express.

[250] Ding H., Su Y., Wei Y. (2025). Off-Axis Illumination to Solve the Forbidden Pitch Problem in Plasmonic Lithography. Opt. Laser Technol..

[251] Verma R., Sharma A., Kargupta K., Bhaumik J. (2005). Electric Field Induced Instability and Pattern Formation in Thin Liquid Films. Langmuir.

[252] Morariu M.D., Voicu N.E., Schäffer E., Lin Z., Russell T.P., Steiner U. (2003). Hierarchical Structure Formation and Pattern Replication Induced by an Electric Field. Nat. Mater..

[253] Schäffer E., Thurn-Albrecht T., Russell T.P., Steiner U. (2001). Electrohydrodynamic Instabilities in Polymer Films. Europhys. Lett..

[254] Park H., Hwang J., Lee J., Kang D.J. (2023). Rapid Electrohydrodynamic-Driven Pattern Replication over a Large Area via Ultrahigh Voltage Pulses. ACS Nano.

[255] Park H., Hwang J., Chae H., Kang D.J. (2024). Rapid In-Plane Pattern Growth for Large-Area Inverse Replication Through Electrohydrodynamic Instability of Polymer Films. Small.

[256] Goldberg-Oppenheimer P., Steiner U. (2010). Rapid Electrohydrodynamic Lithography Using Low-Viscosity Polymers. Small.

[257] Harkema S., Steiner U. (2005). Hierarchical Pattern Formation in Thin Polymer Films Using an Electric Field and Vapor Sorption. Adv. Funct. Mater..

[258] Goldberg-Oppenheimer P., Kohn P., Langford R.M., Steiner U. (2012). Patterning of Crystalline Organic Materials by Electro-Hydrodynamic Lithography. Small.

[259] Rickard J.J.S., Farrer I., Goldberg Oppenheimer P. (2016). Tunable Nanopatterning of Conductive Polymers via Electrohydrodynamic Lithography. ACS Nano.

[260] Zhou Y., Nicolas A., Thomas K.R., Steiner U. (2012). Interplay of Electrohydrodynamic Structure Formation and Microphase Alignment in Lamellar Block Copolymers. Soft Matter.

[261] Boudoire F., Partel S., Toth R., Heier J. (2018). Combining Parallel Pattern Generation of Electrohydrodynamic Lithography with Serial Addressing. RSC Adv..

[262] Busà C., Rickard J.J.S., Chun E., Chong Y., Navaratnam V., Goldberg Oppenheimer P. (2017). Tunable Superapolar Lotus-to-Rose Hierarchical Nanosurfaces via Vertical Carbon Nanotubes Driven Electrohydrodynamic Lithography. Nanoscale.

[263] McCarthy E., Thomas J., Oppenheimer R., Rickard J.J.S., Goldberg P. (2024). Collagen-Electrohydrodynamic Hierarchical Lithography for Biomimetic Photonic Micro-Nanomaterials. Small.

[264] Voicu N.E., Saifullah M.S.M., Subramanian K.R.V., Welland M.E., Steiner U. (2007). TiO_2_ Patterning Using Electro-Hydrodynamic Lithography. Soft Matter.

[265] Kim H.N., Lee S.O., Kang D.J., Lee J.J. (2012). Fabrication of an Inorganic Nano Structure for a Large Area via Electrohydrodynamic Lithography (EHL). J. Nanosci. Nanotechnol..

[266] Lv G., Tian H., Shao J., Yu D. (2022). Pattern Formation in Thin Polymeric Films via Electrohydrodynamic Patterning. RSC Adv..

[267] Wang Q., Wu Q., Li Y., Liu X., Li Y. (2025). Patterning Fidelity Enhancement and Aberration Mitigation in EUV Lithography Through Source–Mask Optimization. Micromachines.

[268] Xu H., Sakai K., Kasahara K., Kosma V., Yang K., Herbol H.C., Odent J., Clancy P., Giannelis E.P., Ober C.K. (2018). Metal-Organic Framework-Inspired Metal-Containing Clusters for High-Resolution Patterning. Chem. Mater..

[269] Ossiander M., Leonidivna Meretska M., Kristin Hampel H., Wei Daniel Lim S., Knefz N., Jauk T., Capasso F., Schultze M. (2023). Extreme Ultraviolet Metalens by Vacuum Guiding. Science.

[270] Päivänranta B., Langner A., Kirk E., David C., Ekinci Y. (2011). Sub-10 Nm Patterning Using EUV Interference Lithography. Nanotechnology.

[271] Zhang Y., Jiang Q., Long M., Han R., Cao K., Zhang S., Feng D., Jia T., Sun Z., Qiu J. (2022). Femtosecond Laser-Induced Periodic Structures: Mechanisms, Techniques, and Applications. Opto-Electron. Sci..

[272] Chen Z., Tu C., Sun H., Kang X., Liu J., Hu S. (2025). Efficient Mask Optimization for DMD-Based Maskless Lithography Using a Genetic–Hippo Hybrid Algorithm. Micromachines.

[273] Esashi M., Kojima A., Ikegami N., Miyaguchi H., Koshida N. (2015). Development of Massively Parallel Electron Beam Direct Write Lithography Using Active-Matrix Nanocrystalline-Silicon Electron Emitter Arrays. Microsyst. Nanoeng..

[274] Meng Y., Peng R., Cheng J., Meng Y., Zhao Q. (2023). Forty-Nanometer Plasmonic Lithography Resolution with Two-Stage Bowtie Lens. Micromachines.

